# Factors affecting the composition of the gut microbiota, and its modulation

**DOI:** 10.7717/peerj.7502

**Published:** 2019-08-16

**Authors:** Nihal Hasan, Hongyi Yang

**Affiliations:** 1 Department of Microbiology, Northeast Forestry University, Harbin, Heilongjiang, China; 2 Faculty of Health Science, Al-Baath University, Homs, Syria

**Keywords:** Antibiotics, miRNA, Diet, Probiotics, Gut microbiota, Prebiotics, AMPs, FMT

## Abstract

Gut microbiota have important functions in the body, and imbalances in the composition and diversity of those microbiota can cause several diseases. The host fosters favorable microbiota by releasing specific factors, such as microRNAs, and nonspecific factors, such as antimicrobial peptides, mucus and immunoglobulin A that encourage the growth of specific types of bacteria and inhibit the growth of others. Diet, antibiotics, and age can change gut microbiota, and many studies have shown the relationship between disorders of the microbiota and several diseases and reported some ways to modulate that balance. In this review, we highlight how the host shapes its gut microbiota via specific and nonspecific factors, how environmental and nutritional factors affect it, and how to modulate it using prebiotics, probiotics, and fecal microbiota transplantation.

## Introduction

The human intestines are inhabited by complex microbial communities called gut microbiota, and the number of these microbes has been estimated to exceed 10^14^. Studies on humans and animal models have reported the role of gut microbiota in human health (De Palma et al., 2017; Wiley et al., 2017). The gut microbiota have many important functions in body, including supporting resistance to pathogens, affecting the immune system (Chung et al., 2012; Arpaia et al., 2013), playing a role in digestion and metabolism (Rothschild et al., 2018), controlling epithelial cell proliferation and differentiation (Sekirov et al., 2010), modifying insulin resistance and affecting its secretion (Kelly et al., 2015), and affecting the behavioral and neurological functions of the host (Buffington et al., 2016; Wahlström et al., 2016; Zheng et al., 2019).

**Figure 1:**
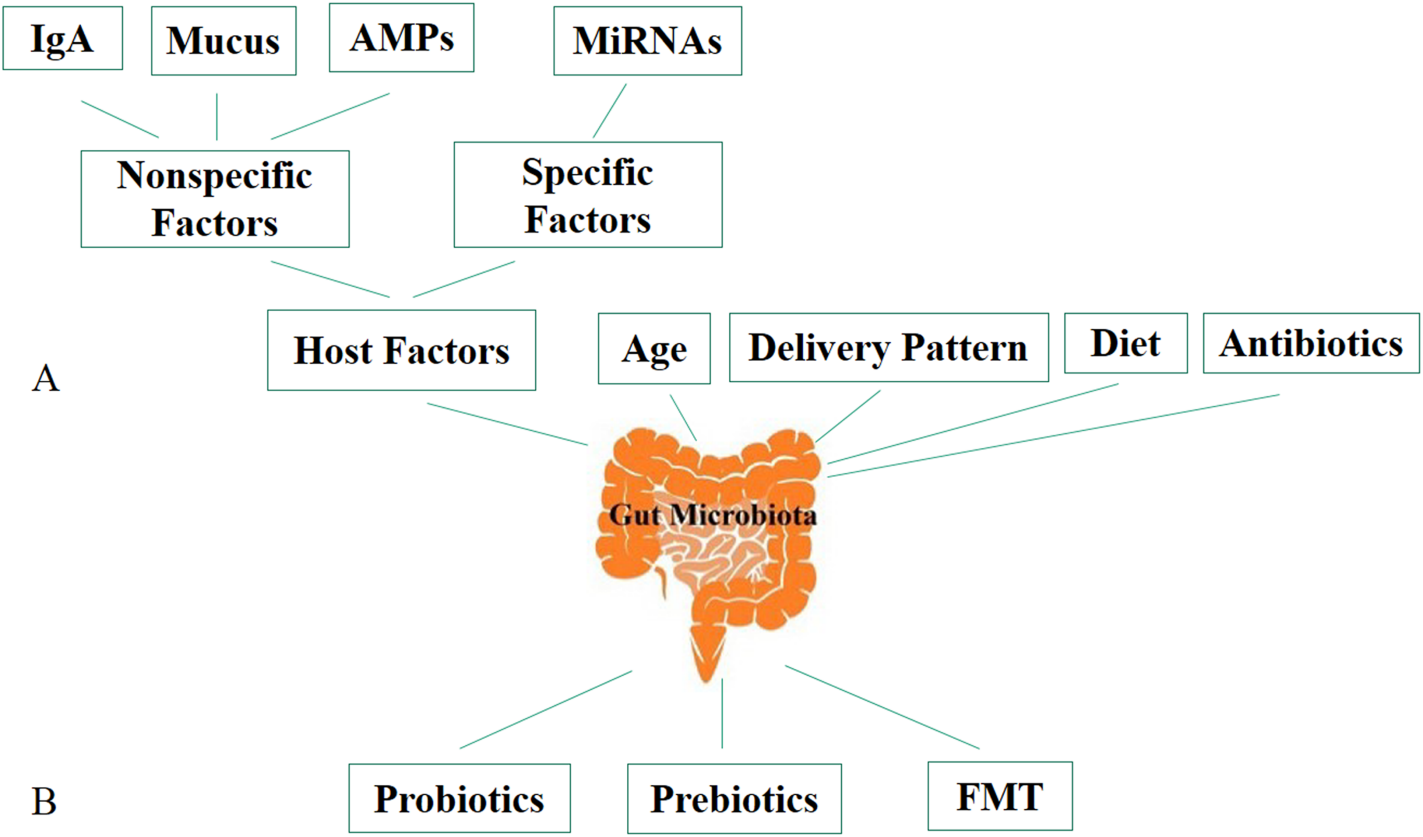
Factors affecting gut microbiota and ways to modulate it. (A) Factors affecting gut microbiota. (B) Ways to modulate gut microbiota. AMPs, antimicrobial peptides; IgA, immunoglobulin A; miRNA, microRNA; FMT, fecal microbiota transplantation.

Studies have shown that transplantation of gut microbiota of the conventional zebrafish to germ-free mice and conventional mouse gut to germ-free zebrafish resulted the gut microbiota of both receivers after transplantation resembled the microbiota of their conventional species. It indicates that there are specific mechanisms used by the host to shape its own gastrointestinal microbiota and maintain its homeostasis (Liu et al., 2016; Rawls et al., 2006). There are several factors that can change gut microbiota, including host genetic, diet, age (Odamaki et al., 2016; Jandhyala et al., 2015), mode of birth (Nagpal et al., 2017; Wen & Duffy, 2017) and antibiotics (Goodrich et al., 2014; Ley et al., 2005; Turnbaugh et al., 2009) (Fig. 1A). The perturbation of the gut microbiota population associated with several human diseases that include inflammatory bowel diseases (IBD) (Bien, Palagani & Bozko, 2013; Takahashi et al., 2016; Nishino et al., 2018), obesity and diabetes (Karlsson et al., 2013), allergy (Vernocchi, Del Chierico & Putignani, 2016; Bunyavanich et al., 2016), autoimmune diseases (Chu et al., 2017), cardiovascular disease (Cui et al., 2017b; Jie et al., 2017; Trøseid, 2017), hypertension (Li et al., 2017), and modulating its composition and diversity is considered a promising treatment for these diseases. There are many ways to modulate gut microbiota, including probiotics, prebiotics, and fecal microbiota transplantation (FMT) (Fig. 1B), which can cause favorable changes in the structure and functions of the gut microbiota and restore it temporarily or permanently. The present review discusses the mechanisms the host uses to shape its gut microbiota and the factors that can modulate these microbes and to improve understanding of the role of FMT, probiotics, and prebiotics in gut community modulation.

## Survey methodology

Literature searching aimed at collecting any published data about cultivation, alteration, and restoration of gut microbiota. We searched literature relevant to the topic of the articles using PubMed and Google Scholar. Key words such as gut microbiota, factors shape gut microbiota, microRNA (miRNA), FMT, probiotics, antibiotic, prebiotics, and antimicrobial peptides (AMPs) were used to search. Then, screened articles were used as references for this review.

### Host factors that shape the human microbiota

Hosts use specific and nonspecific factors to cultivate their own gut microbiota. The host favors the type of microbes that can colonize its intestines and removes other microbes from the body.

### Nonspecific host factors

The host chooses its own gut microbiota by producing several molecular signals that control the structure of the surfaces colonized by microbiota and so influences its composition. These molecules are produced by the intestinal epithelial cells (IEC) and include mucus, AMPs, and immunoglobulin A (IgA), which can encourage the growth of some microbial species and inhibit that of others. In the large intestine, mucus plays an important role in keeping the microbes far from IEC, it is constituted of two layers: the inner layer does not contain any microorganisms whereas the outer layer contains soluble mucins, which are decorated by O-glycans, provide a nutrient source and binding site for gut microbiota (Podolsky et al., 1993; Artis et al., 2004). Mucus and mucin O-glycans play a key role in shaping the gut microbiota and selecting the most appropriate microbial species for host health (Arpaia et al., 2013; Zarepour et al., 2013). Utilisation of mucin by gut microbiota is depending on glycoside hydrolases and polysaccharide lyases which are encoded by their genes (Tailford et al., 2015). Some species of gut microbiota have capacity to degrade complex carbohydrates such as *Bacteroides thetaiotaomicron*, whose genome contains 260 glycoside hydrolases (Cockburn & Koropatkin, 2016). While there is not enough mucus in the small intestine, AMPs play an important role in shaping gut microbiota. The gut microbiota by its structural components and metabolites induce AMP production by Paneth cell through a mechanism mediated by pattern recognition receptor (PRR) (Hooper, 2009; Salzman, Underwood & Bevins, 2007), whereas PRRs are activated by different microbial components, such as flagella and lipopolysaccharide, in a system called microbe-associated molecular patterns (MAMP). PRR-MAMP plays important roles in promoting the function of the mucus barrier and inducing the production of IgA, mucin glycoproteins, and AMPs (Takeuchi & Akira, 2010; Carvalho et al., 2012), and concentration of the AMPs are maximal in small intestinal crypts because of Paneth cells reside at this location. AMPs are secreted by IEC as the body's first line of defense against invaders and these proteins have a broad effect that directly kills bacteria, virus, yeast, fungi, and even cancer cells. These proteins include defensins, cathelicidins, Reg family proteins, and ribonucleases (Cash et al., 2006). It has been proven that all dominant species of gut microbiota can resist high concentrations of AMPs, and AMPs may play an important role when the host distinguishes commensal from pathogenic bacteria (Cullen et al., 2015). *Bacteroides*, the most abundant Gram-negative genus among the gut microbiota, had resistance to AMPs (Cullen et al., 2015). After *Bacteroides* strains lost their ability to produce dephosphorylate lipid A (Raetz & Whitfield, 2002), the sensitivity to AMPs is increased, suggesting that dephosphorylate lipid A gives the *Bacteroides* their ability to resist the high concentrations of AMPs in the intestine.

Antibacterial lectins, which can form hexameric pores in Gram-positive bacterial membranes and prevent them from reaching the intestinal mucus layer (Cash et al., 2006; Mukherjee et al., 2014), are important antibacterial factors that contribute to shaping gut microbiota. On the basis of these secreting factors, IEC promote growth of some bacterial species and inhibit that of others so the host control the shape and structure of its gut microbiota by modulating these secretors.

In addition, there are plasma cells in the intestinal mucosa that produce secretory immunoglobulin A (SIgA) that can cover the bacteria and control its numbers locally (Macpherson & Uhr, 2004). Further, SIgA plays a role in bacterial biofilm formation by binding to SIgA receptors on bacteria (Randal Bollinger et al., 2003). The presence of gut microbiota was possibly a condition to activate dendritic cells, which induce plasma cells to produce IgA (Massacand et al., 2008). The absence of IgA can lead to increases in segmental filamentous bacteria, occurs IgA-deficient mice, suggesting that the induced secretory IgA production is a form of competition between different types of gut microbiota (Suzuki et al., 2004).

### Specific host factors

The other host factor that can be used to assess the shape and structure of gut microbiota is miRNAs, which are small non-coding RNAs that are 18**–**23 nucleotides in length. MiRNAs are generated in the nucleus and then transported to the cytoplasm to effect gene silencing by hybridizing with the 3′ untranslated region of the target gene and promote mRNA degradation or inhibit translation (Djuranovic, Nahvi & Green, 2012). One miRNA can target different mRNAs (Maudet, Mano & Eulalio, 2014; Kalla et al., 2014), and it has been proven that miRNAs exist outside the cells and circulate in bodily fluids (Weber et al., 2010).

Ahmed et al. (2009) and Link et al. (2012) measured RNA in human stool and demonstrated that miRNAs as potential markers of intestinal malignancy, whereas Liu et al. (2016) investigated the gut miRNAs in intestinal contents and feces. All three works demonstrated their ability to affect the composition of the gut microbiota composition. The IEC and Hopx-positive cells are main sources of fecal miRNA. Ahmed et al. (2009) and Link et al. (2012) also investigated the relationship between the deficiency of IEC-miRNA and gut dysbiosis and studied why wild-type fecal transplantation can restore the gut microbiota, indicating that miRNAs were able to regulate the gut microbiome. Several miRNAs can then enter the gut bacterial cells and regulate their growth and gene expression. Liu et al. (2016) found that hsa miRNA-515-5P can stimulate *Fusobacterium nucleatum* growth and that hsa miRNA-1226-5p stimulates *E. coli* growth via culturing the strains with synthesized mimics miRNA in vitro and affected *E. coli* in vivo also when given orally to mice. In this way, miRNA was found to have specific effects on gut bacterial growth.

In addition, Liu et al. (2016) demonstrated that miRNA can enter the bacterial cell and settle near bacteria DNA in the nucleus, and then, they studied the effect of synthetic miRNAs on bacterial gene expression and growth. The presence of gut microbiota has an effect on miRNA expression in the intestine. Dalmasso et al. (2011) found that nine miRNAs in the colon and ileum had different levels of expression in the germ-free mice which are colonized with pathogen-free mice microbiota. Xue et al. (2011) found that microbiota can affect expression of miR-10a, which targets interleukin-12/ interleukin-23p40B, which is itself responsible for innate immune response of the host toward the gut microbiota, suggesting that the gut microbiota can control host innate immune responses by regulating of miRNA expression.

The gut microbiota can be shaped by administration of fecal miRNAs. The microbiota profile of the recipient becomes similar to that of the donor after transplantation of fecal RNA, as shown in the case of transfer from healthy mouse donors to IEC-deficient mice (Liu et al., 2016). This indicates an important potential of application, in the future, diseases related to changes in gut microbiota may be treated using synthetic specific miRNA.

### Factors influencing homeostasis of microbiota

The gut microbiota is established early in life stage but can later be altered by various factors that affect its development and diversity.

### Age and delivery pattern

Microbial intestinal colonization process begins in utero by microbiota in the amniotic fluid and placenta (Collado et al., 2016). Studies have reported that there are bacteria and bacterial products, such as DNA, in meconium (Nagpal et al., 2017; Wampach et al., 2017; Jiménez et al., 2008), amniotic fluid (Collado et al., 2016; DiGiulio et al., 2008), and the placenta (Collado et al., 2016; Friedrich, 2013). Studies on the pregnant mice have demonstrated that oral administration of labeled *Enterococcus fecium* resulted staring isolated it from the newborn stool samples (Jiménez et al., 2008), this result agree with the evidence maternal microbes can be transferred to the amniotic fluid (Collado et al., 2016; Jiménez et al., 2008) and the placenta (Goldenberg, Hauth & Andrews, 2000). After birth, the mode of delivery affect the early-life development of the gut microbiota. Newborns delivered vaginally have primary gut microbiota dominated by *Lactobacillus* and *Prevotella* derived from the mother's vaginal microbiota, while those born via cesarean delivery derive their gut microbiota from the skin, leading to dominance of *Streptococcus*, *Corynebacterium*, and *Propionibacterium* (Dominguez-Bello et al., 2010; Mackie, Sghir & Gaskins, 1999). These primary microbiota evolve over time to become more diverse and relatively stable. At the age 3 years, they become similar to an adult's gut microbiota (Yatsunenko et al., 2012).

### Diet

After birth, the first effect on the gut microbiota is the infant diet (breast or formula milk). The composition of the milk affect on shaping the early gut microbiota (Guaraldi & Salvatori, 2012; Groer et al., 2014). In breast feeding infants, the species that dominate the gut microbiota are *Lactobacillus* and *Bifidobacterium*; breast milk contains oligosaccharides that can be broken down easily by these species, resulting in an increase in short-chain fatty acids, which directs the immune system to increase the expression of immunoglobulin G (Ouwehand, Isolauri & Salminen, 2002). Whilst in infants raised on formula, the dominant species are *Enterococcus*, *Enterbacteria*, *Bacteroides*, *Clostiridia*, and *Streptococcus* (Yoshioka, Iseki & Fujita, 1983; Stark, Lee & Parsonage, 1982). The primary microbiota acquired during infancy may play an important role in initial immunity during the growth of babies, for this reason, the composition of primary microbiota during this period is very important to protect babies from diseases related to poor immunity (Groer et al., 2014; Sherman, Zaghouani & Niklas, 2014). Several studies have compared the gut microbiota population and mucosal immune response between breast and formula feeding and found that the breast feeding caused more stable population and good mucosal immune response (Grönlund et al., 2000; Bezirtzoglou, Tsiotsias & Welling, 2011).

Interestingly, human milk microbiota play important roles in immunological activities such as increasing the number of plasma cells in the intestinal environment of newborns that produce IgA (Gross, 2007), stimulation of cytotoxic Th1 cell (M'Rabet et al., 2008), motivation specific cytokines that create a balanced microenvironment (Walker & Iyengar, 2014), development of local and systemic immune system (Houghteling & Walker, 2015), and some *Lactobacillus* strains promote the production of Th1, cytokines and TNF-α, regulate T cell, and stimulate NK cells, CD4+ T-cells and CD8+ T-cells.

After infancy, the gut microbiota continues its development, and the diet remains key to defining the shape, structure, and diversity of the gut microbiota. Vegetarian diets have been found to be associated with healthy, diverse gut microbiota characterized by the domination of species that can metabolize insoluble carbohydrates, such as *Ruminococcus*, *Roseburia*, and *Eubacterium* (Walker et al., 2011), while a non-vegetarian diet (Western diet) has been associated with a decreasing number of *Firmicutes* and an increase in *Bacteroides* (David et al., 2014). Diet can cause important changes even over short periods (David et al., 2014). After consumption of a Western diet, the gut microbiota ferment amino acids, which result in production of short-chain fatty acids as energy sources, and harmful compounds can be produced (Windey, De Preter & Verbeke, 2012). The vegetarian diet inhibits this, and fosters carbohydrate fermentation as the main function of microbiota (Tang et al., 2013; Clarke et al., 2014).

### Antibiotics

The use of antibiotics is a two-edged weapon: it destroys both pathological and beneficial microbes indiscriminately, allowing loss of gut microbiota or the so-called dysbiosis and growth of unwanted microbes (Klingensmith & Coopersmith, 2016). Studies on experimental mice have demonstrated the antibiotics administration affected secondary bile acid and serotonin metabolism in the colon and resulting in delayed intestinal motility by causing microbiota depletion (Ge et al., 2017). Antibiotics disrupt the competitive exclusion machinery, a basic property by which microbiota eliminate pathological microbes (Hehemann et al., 2010). This disruption promotes growth of other pathogens, such as *Clostridium difficile* (Ramnani et al., 2012). Studies have reported that clindamycin (Jernberg et al., 2007), clarithromycin and metronidazole (Jakobsson et al., 2010) and ciproflaxin (Dethlefsen & Relman, 2010) affect the microbiota structure for a long time.

Clindamycin causes changes in microbiota that last 2 years with no recovery in the diversity of *Bacteroides* (Jernberg et al., 2007). Similarly, the use of clarithromycin in *Helicobacter pylori* treatment causes a decrease in the population of *Actinobacteria* (Jakobsson et al., 2010), while ciprofloxacin has been shown to cause a decrease in that of *Ruminococcus* (Dethlefsen et al., 2008). Vancomycin, which is considered the best treatment option for *C. difficile* infection (CDI), but, like other antibiotics, it causes changes in gut microbiota that lead to recurrent *C. difficile* infection (rCDI) (Zar et al., 2007) or encourage growth of pathological strains of *E. coli* (Lewis et al., 2015). Vancomycin treatment also causes depletion of most gut microbiota, such as *Bacteroidetes*, and it is associated with increases in *Proteobacteria* species (Isaac et al., 2016), and decreases in *Bacteroidetes*, *Fuminococcus*, and *Faecalibacterium* (Vrieze et al., 2014). The specific effects of antibiotic administration on the gut microbiota and the recovery time are depending on individual, like effect of the changes in the gut microbiota before treatment (Dethlefsen & Relman, 2010; Jernberg et al., 2007).

In many countries, the antibiotics are used for agriculture particularly intensive farming of poultry and beef, and low doses of antibiotics are routinely given to livestock to increase their growth and weight (Blaser, 2016). Several studies on human and rodent have indicated to an obesogenic impact of antibiotics in humans even in low doses found in food (Blaser, 2016). Pesticides and other chemicals are commonly sprayed on foods and currently, the evidence is lacking for their harm on gut health and the effects of organic food (Lee et al., 2017).

### Modulation of gut microbiota

Modulation of the gut microbiota is clinically important to treat all diseases related to imbalances in gut microbiota, and methods for modulating that balance include probiotics, prebiotics, and FMT.

### Probiotics

Probiotics are living microorganisms that, when taken in appropriate doses, protect human health (Kristensen et al., 2016). The most commonly used probiotic species are *Lactobacillus*, *Bifidobacteria* and yeasts, such as *Saccharomyces boulardii* (Rokka & Rantamäki, 2010; Dinleyici et al., 2012). The proposed mechanisms by which probiotics improve health include promotion of favorable types of gut microbes. In a study by Stratiki et al. (2007), the administration of a Bifidobacter-supplemented infant formula decreased the intestinal permeability of preterm infants and increased the abundance of fecal *Bifidobacterium*. Probiotics compete with harmful species for adhesion sites. For example, *E. coli* Nissle can move and attack pathological microbes and prevent their adhesion (Servin, 2004). Some probiotics can produce antimicrobial compounds like *Lactobacillus reuteri*, which produce reuterin that kills harmful microbes directly (Cleusix et al., 2007), and induces immune responses in the host. *Bifidobacterium* can enhance the function of mucous intestinal barrier, increase serum IgA, and reduce inflammation of the intestine (Mohan et al., 2008). *Bifidobacterium* has also been reported to reduce the number of harmful bacteria in stool samples (Mohan et al., 2006). Several systematic reviews analyzed the role of probiotics on clinical outcomes. The analysis showed substantial evidence for beneficial effects of probiotic supplementation in chronic periodontitis (Ikram et al., 2018), urinary tract infections (Schwenger, Tejani & Loewen, 2015), necrotizing enterocolitis (Rees et al., 2017), and reduction of total cholesterol and low-density lipoprotein cholesterol (Wu et al., 2017). In type 2 diabetes patients, probiotic administration reduced fasting blood glucose and HbA1 (Akbari & Hendijani, 2016) and decreased cardiovascular risk (Hendijani & Akbari, 2018). A meta-analysis showed that probiotic therapy for patients with type 2 diabetes significantly decreased insulin concentration and homeostasis model assessment of insulin resistance score (Zhang, Wu & Fei, 2016), which was an important index to assess insulin resistance in several diseases, such as prediabetes, diabetes mellitus type 2 and metabolic syndrome. Xie et al. (2017) reported the efficiency of probiotics in vulvovaginal candidiasis in non-pregnant women indicated that probiotics increased the rate of short term clinical cure and mycological cure and decreased relapse rate at 1 month.

The utilization of mucins by gut microbiota under dietary fiber deficiency causes the erosion of the colonic mucus barrier (Desai et al., 2016) and studies on experimental mice demonstrated that administration of *Akkermansia muciniphila*, mucin degrade, lead to restoration of the gut barrier (Everard et al., 2013; Li et al., 2016a). *A. muciniphila* has probiotic properties and can prevent or treat several metabolic disorders (Zhou, 2017). Recently, studies on chow diet-fed mice showed that *A. muciniphila* supplementation had ability to reduce metabolic inflammation that lead to alleviating body weight gain and reduce fat mass (Zhao et al., 2017). Moreover, *A. muciniphila* acts as an energy sensor, its abundance increases with decreasing calories and decreases with extra energy which enables the energy uptake when available (Chevalier et al., 2015).

Many probiotic products use the microencapsulation technique to protect bacteria from environmental factors, and their effects vary depending on the type and number of bacteria (Rokka & Rantamäki, 2010). Generally, the probiotic products contain at least 10^6^**–**10^7^ CFU. The different doses of probiotics in treating antibiotic**-**induced diarrhea were compared, indicating that high doses of probiotics were more effective (Gao et al., 2010). This is because a large number of the probiotic organisms perish in the stomach before reaching the colon (Borody & Campbell, 2011).

Currently, researchers do not agree on the efficacy of probiotics in intestinal diseases, largely because studies have used different doses and types of probiotics (Ringel & Ringel-Kulka, 2011; Hamilton et al., 2012; Borody et al., 2004). For example, some studies have shown that probiotics mitigate traveler's diarrhea while others have found them to have no clear benefit (Ritchie & Romanuk, 2012; Huebner & Surawicz, 2006).

### Prebiotics

Prebiotics are defined as selective fermentation components that cause specific changes in the activity or structure of gut microbiota and so benefit to the host (Tabibian, Talwalkar & Lindor, 2013; Rossen et al., 2014). Prebiotics usually consist of non-digestible carbohydrates, oligosaccharides, or short polysaccharides like inulin, oligofructose, galactofructose, galacto-oligosaccharides, and xylo-oligosaccharides. Prebiotics must be able to resist gastric acids but yet be degraded by digestive enzymes and become absorbed by the upper tract of the digestive system, fermentation by gut microbiota and inciting the growth or activating useful species of the gut microbiota (Quraishi et al., 2014). Prebiotics affect the microbiota species already present in the colon (Slavin, 2013). The main target species of prebiotics are *Lactobacilli* and *Bifidobacteria* (Tabibian, Talwalkar & Lindor, 2013) and the effects of prebiotics include increasing production of short-chain fatty acids and decreasing pH (De Vrese & Marteau, 2007).

Dietary fiber consumption is important to maintain intact mucosal barrier function in the gut (Ray, 2018). Recently, several studies demonstrated the effect of prebiotic fibers on the modulation of the gut microbiota (Dahiya et al., 2017; Gibson et al., 2017; Holscher, 2017; Umu, Rudi & Diep, 2017; Ling et al., 2016; Hu et al., 2016). Studies showed that administration of inulin can prevent the harmful effects of high fat diets on penetrability of the mucus layer and metabolic functions (Zhou, 2017; Schroeder et al., 2018). Evidence indicates that the low fiber Western diet causes weakening in the colonic mucus barrier, that leads to microbiota crept, which results in pathogen susceptibility (Desai et al., 2016) and inflammation (Earle et al., 2015), providing a relationship between the Western diet with chronic diseases. Moreover, the mixture of galacto-oligosaccharides and fructo-oligosaccharides have ability to increasing *Bifidobacteria* and decreasing *Clostridium* in the gut whilst galacto-oligosaccharides alone increase *Lactobacillus* (Vandenplas, Zakharova & Dmitrieva, 2015). Additional studies showed that arabino-xylooligosaccharides and inulin alter the intestinal barrier function and immune response (Van den Abbeele et al., 2018).

### FMT

**Figure 2:**
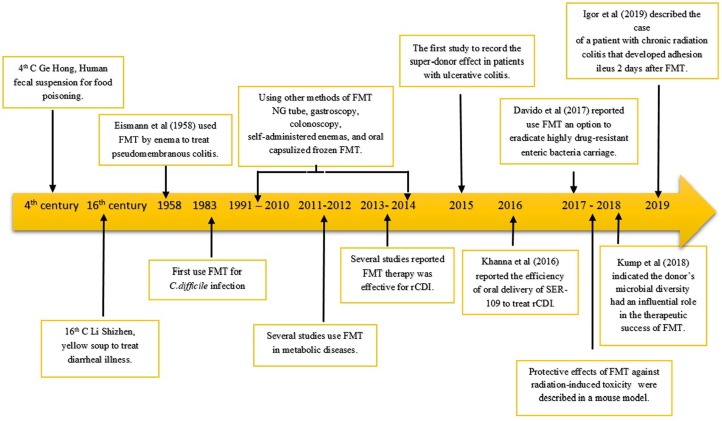
Timeline: FMT development studies. Yellow arrow showed time-base, and FMT development studies were described in text box.

Fecal microbiota transplantation is the process of transplantation of fecal bacteria from healthy donors to patients with intestinal diseases or changes or dysbiosis in natural gut microbiota to restore the community and function of gut microbiota (Khoruts & Sadowsky, 2016). The first use of FMT took place in China during the 4th century (Fig. 2). Ge Hong reportedly used faeces to treat cases of food poisoning and diarrhea (Zhang et al., 2012). In the 16th century, Li Shizhen reportedly used fecal matter, later called yellow soup, to treat fever, pain, and several symptoms of intestinal disorders, such as diarrhea, constipation, and vomiting. In modern medicine, Eiseman first reported that FMT has successfully treated many patients with pseudomembranous colitis by enema (Eiseman et al., 1958). In the succeeding years, the use of FMT focused on the treatment of CDI and rCDI with a cure rate of 87**–**90% (Kassam et al., 2012; Van Nood et al., 2013; Cammarota, Ianiro & Gasbarrini, 2014; Rossen et al., 2015). Currently, because of insufficient genetic information that explains the contrast in recipient responses, investigators have studied the donor-dependent effect and suggested the existence of so-called super-donors. In 2015, Moayyedi and colleagues described the super-donor effected on the efficacy of FMT for inducing clinical remission in patients with ulcerative colitis (Moayyedi et al., 2015). Whilst Kump et al. (2018) and Vermeire et al. (2015) demonstrated that the donor's microbial diversity has an influential role in the therapeutics success of FMT (Fig. 2).

Fecal microbiota transplantation has a high capacity for treating different diseases, including IBD (Ianiro et al., 2014; Anderson, Edney & Whelan, 2012; Goyal et al., 2018; Kump et al., 2018), irritable bowel syndrome (Pinn, Aroniadis & Brandt, 2014; Gibson & Roberfroid, 1995; Borody et al., 2004; Holvoet et al., 2017; Mizuno et al., 2017; Aroniadis et al., 2018; Halkjær et al., 2018; Johnsen et al., 2018) constipation (Tian et al., 2017; Ding et al., 2017), metabolic diseases (Tremaroli & Bäckhed, 2012; Tilg & Kaser, 2011; Vrieze et al., 2012), neuropsychiatric conditions (Hornig, 2013; He et al., 2017; Kang et al., 2017; Makkawi, Camara-Lemarroy & Metz, 2018), autoimmune diseases (Luckey et al., 2013; Borody et al., 2011), allergic disorders (Russell & Finlay, 2012), blood (Kakihana et al., 2016; Spindelboeck et al., 2017; DeFilipp et al., 2018), colon cancer (Wong et al., 2017), anti-tumor immunity (Sivan et al., 2015) and chronic fatigue syndrome (Borody, Nowak & Finlayson, 2012). Some studies using FMT to treat CDI and IBD are summarized in Table 1. In the most recent studies, Davido et al. (2017) reported to eradicate highly drug-resistant enteric bacteria using FMT. While Cui et al. (2017a) and Gerassy-Vainberg et al. (2018) reported the protective effects of FMT against radiation-induced toxicity in a mouse model. Recently, a non-desirable side effect of the FMT was reported. Harsch & Konturek (2019) reported that a patient with chronic radiation colitis developed adhesion ileus 2 days after FMT (Fig. 2).

**Table 1:** Summary of studies using FMT to treat CDI and IBD.

Administration method	Resolution rate (%)	*n*	Diagnosis	References
Colonoscopy	100	1	CDI	Persky & Brandt, 2000
Nasogastric	83	18	CDI	Aas, Gessert & Bakken, 2003
Colonoscopy, enema	93.7	16	CDI	Wettstein et al., 2007
Rectal catheter	97.7	45	CDI	Louie et al., 2008
Colonoscopy	100	1	CDI	Hellemans, Naegels & Holvoet, 2009
Colonoscopy	92	37	CDI	Arkkila et al., 2010
Self-administered enema	100	7	CDI	Silverman, Davis & Pillai, 2010
Colonoscopy	100	13	CDI	Wilcox, 2011
Colonoscopy	94	70	CDI	Mattila et al., 2012
Colonoscopy	86	43	CDI	Hamilton et al., 2012
Nasogastric	80	74	CDI	Rubin et al., 2013
Nasoduodenal tube	81	17	CDI	Van Nood et al., 2013
Enema	33	10	IBD	Kunde et al., 2013
Colonoscopy	20	5	IBD	Damman et al., 2014
Oral capsule	70	20	CDI	Youngster et al., 2014b
Colonoscopy	90	20	CDI	Cammarota et al., 2015
Various	90–97.8	1,029	CDI	Rossen et al., 2015
Nasogastric infusion	86.6	30	IBD	Cui et al., 2015
Nasogastric infusion	78	9	IBD	Suskind et al., 2015
Enema	30	81	IBD	Paramsothy et al., 2016
Colonoscopy	90.9	46	CDI	Kelly et al., 2016
Colonoscopy	70	30	IBD	Uygun et al., 2017

**Note:**

CDI, *Clostridium difficile* infection; IBD, inflammatory bowel disease; *n*, number of patients.

### Transplantation pathway of FMT

**Figure 3:**
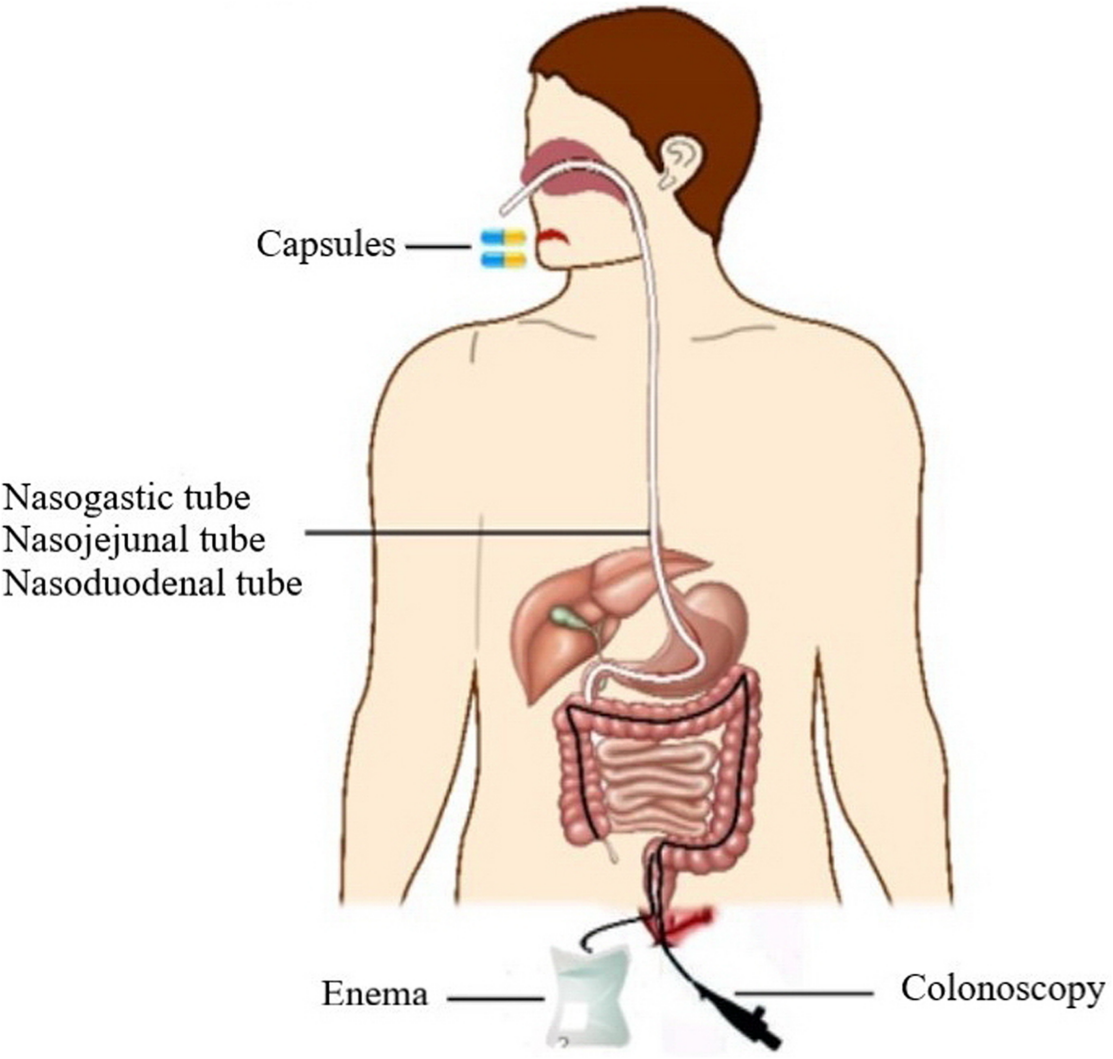
The pathways of FMT. Several pathways of FMT are shown in the schematic diagram.

Several pathways (Fig. 3), such as colonoscopy, and enema, have shown pronounced efficiency in FMT (Paterson, Iredell & Whitby, 1994; Borody et al., 2003; Silverman, Davis & Pillai, 2010; Kassam et al., 2012). More recently, enteric-coated capsules and capsules containing freeze dried feces or bacteria were also used and the high efficiency of FMT was confirmed (Brandt, Borody & Campbell, 2011; Tian et al., 2015).

Enema is still the easiest, cheapest way of effecting FMT, posing little risk to patients. Recently, colonoscopy has become an attractive option because it can deliver a large volume of fecal matter along the entire colon. However, it is less safe than rectal enemas, posing a greater risk of perforation (Kassam et al., 2013). Colonoscopy is also more expensive and time-consuming than enema.

Many recent studies have reported that the stool can be delivered via oral encapsulated FMT or stool enemas (Youngster et al., 2014a; Hirsch et al., 2015). The efficacy of the first dose was about 52**–**70%, which is lower than that of other methods of administration, such as colonoscopy; but after examination of several rounds of treatment are administered to the patient, the result were similar to those of colonoscopy (Youngster et al., 2014a; Hirsch et al., 2015).

SER-109, a microbiome therapeutic, is a new kind of therapy composite of commensal bacterial spores. Studies have shown that SER-109 not only relieves dysbiosis but also leads to increases in beneficial bacteria not contained in the product when treating rCDI in patients with a history of multiple relapses (Khanna et al., 2016). Several studies have also shown that spore-forming organisms isolated from stool sample, could compete metabolically with *C. difficile* for essential nutrients (Buffie et al., 2015; Theriot et al., 2014), and this could prevent CDI. Whilst Khanna et al. (2016) reported the efficiency of oral delivery of SER-109 to treat rCDI.

Regardless of the route of FMT, there is enough evidence supporting the conclusion that FMT is a highly efficient, and therapeutic option for several intestinal diseases, characterized by ability to restore the compositions and functions of gut microbiota which are similar to gut microbiota of recipients (Khoruts et al., 2010; Li et al., 2016b). Using next**-**generation sequencing techniques, changes in gut microbiota species could been identified both before and after FMT. An increasing studies have shown that FMT can lead to permanent participation of new species of gut microbiota, such as that found in healthy donor feces, and FMT can increase the number of different bacteria present in recipient feces (Khoruts et al., 2010). Currently, the safety problem of FMT is a main obstacle to use it in more patients because of the complexity of feces microbial community.

### Comparison of the efficiency of FMT and probiotics

Fecal microbiota transplantation has been found to outperform probiotics. Probiotic treatments contain specific bacteria and there is some doubt whether a sufficient quantity survives the stomach acids to reach the gut. To become established in the gut, the probiotic strains must outcompete the resident microbiota and occupy an ecological niche. Because the resident microbiota is inherently resistant to outside colonizer (Stecher & Hardt, 2008), the probiotic strains may fail to colonize and function in the gut. In addition, probiotics restore microbiota for an interim period (Tannock et al., 2000). FMT treatment delivered plenty of fecal bacteria to the colon and caused permanent restoration of microbiota (Grehan et al., 2010). Cammarota, Ianiro & Gasbarrini (2014) found that FMT administered to the lower intestinal track was more effective with rCDI than delivery to the upper tract. This may be because the growth of many kinds of fecal microbiota is affected by their administration route, for example, *Bacteroidetes* is sensitive to digestive acids, and the lower tract offers it a gentler environment (Burns, Heap & Minton, 2010). Probiotics rarely remain in the colon more than 14 days after the patient ceases taking them, while the effects of FMT on the gut microbiota can persist for 24 weeks (Pepin et al., 2005; Kelly & LaMont, 2008), indicating that FMT can cause pronounced changes in the gut microbiota (Mattila et al., 2012).

## Future work

Several studies have investigated the role of gut microbiota in many diseases, including colorectal cancer, liver disease, and others (Dicksved et al., 2009; Yang et al., 2009; Claud et al., 2013; Couturier-Maillard et al., 2013; Udayappan et al., 2014; Llopis et al., 2014; Buie, 2015). In the last few years, gut microbiota has been studied using metagenomic methodologies (Qin et al., 2010). Zheng et al. (2019) reported that the gut microbiome from patients with schizophrenia might be relevant to pathology of schizophrenia via altering neurochemistry and neurologic function. Currently, methods of modulating gut microbiota, including FMT, probiotics, and prebiotics, have come to be considered suitable treatment options for these diseases.

Future studies of probiotics and prebiotics should focus on the effect on different diseases with an agreement regarding the doses and the kind of bacteria used under each set of pathological conditions. High-throughput technologies allow researchers to easily find answers to many questions surrounding probiotics and prebiotics. This may help us to design new probiotics that more efficient with a higher quality and may lead to find new bacterial strains with probiotics properties.

Many studies have confirmed the ability of the gut microbiota to modify the expression of the host genes, and the impact of microbiota on miRNA expression was discovered using miRNA arrays, supporting the fact that gut microbiota affects the expression of hundreds of genes in the host (Dalmasso et al., 2011; Watson & Hall, 2013). More studies are needed to understand miRNA**-**microbiota interactions and determine which kinds of microbiota can modulate miRNA expression by combining high-throughput technologies.

Recently, FMT has been found to be a perfect treatment for rCDI cases that are nonresponsive to antibiotic therapy. Its high efficiency in many diseases has been confirmed, but FMT treatment suffers from a lack of information about the safety of long- and short-term administration. More and safer synthetic bacteria products (e.g., encapsulated formulations and full-spectrum stool-based products) and methods of transplantation need be developed to make FMT easier to use and more acceptable to patients. SER109, the mixture of bacterial spores, has shown high efficiency with rCDI diarrhea, and other similar products are being developed. The synthetic bacteria products may be able to replace traditional fecal treatment. In the future, various bacterial products may contain complicated mixtures of different bacterial species tailored using microbial ecological principles, and doctors may choose the suitable synthetic bacteria mixtures to a specific disease. FMT, probiotics, and other treatments meant to modulate a healthy microbiota may come to be considered suitable alternative treatments to antibiotics for diseases related to imbalances of gut microbiota.

## Conclusions

Maintaining gut microbiota homeostasis is very important to the health. Several factors can directly or indirectly effect on the gut microbiota composition and species abundance. Other factors, different from that mentioned in this review, such as geographical location, maternal lifestyle (urban or rural), and fetal swallowing amniotic fluid (Thursby & Juge, 2017; Chong, Bloomfield & O'Sullivan, 2018; Nagpal et al., 2017), are also probable to contribute to gut microbiota development. The disorder of the gut microbiota is associated with several diseases and manipulating its composition and diversity is important element to control the development of these diseases. Based on data published, it can be concluded that manipulating the gut microbiota, either by prebiotics, probiotics or FMT, seem to be attractive options for the prevention and treatment of disease.

However, further studies need a full understanding on how manipulating gut microbiota can enhance health. In addition, studies for detecting the missing functions in the gut microbiota during disease will help to select the potential prebiotic, probiotic, or FMT that achieve the desired benefit.

## Additional Information and Declarations

### Data Availability

The following information was supplied regarding data availability: This is a review article; no data was collected.

## References

[ref-1] Aas J, Gessert CE, Bakken JS (2003). Recurrent *Clostridium difficile* colitis: case series involving 18 patients treated with donor stool administered via a nasogastric tube. Clinical Infectious Diseases.

[ref-2] Ahmed FE, Jeffries CD, Vos PW, Flake G, Nuovo GJ, Sinar DR, Naziri W, Marcuard SP (2009). Diagnostic microRNA markers for screening sporadic human colon cancer and active ulcerative colitis in stool and tissue. Cancer Genomics-Proteomics.

[ref-3] Akbari V, Hendijani F (2016). Effects of probiotic supplementation in patients with type 2 diabetes: systematic review and meta-analysis. Nutrition Reviews.

[ref-4] Anderson JL, Edney RJ, Whelan K (2012). Systematic review: faecal microbiota transplantation in the management of inflammatory bowel disease. Alimentary Pharmacology & Therapeutics.

[ref-5] Arkkila PE, Uusitalo-Seppälä R, Lehtola L, Moilanen V, Ristikankare M, Mattila EJ (2010). 29 Fecal bacteriotherapy for recurrent *Clostridium difficile* infection. Gastroenterology.

[ref-6] Aroniadis OC, Brandt LJ, Oneto C, Feuerstadt P, Sherman A, Wolkoff AW, Downs IA, Zanetti-Yabur A, Ramos Y, Cotto CL, Kassam Z, Elliott RJ, Rosenbaum R, Budree S, Sadovsky RG, Timberlake S, Swanson P, Kim M, Keller MJ (2018). 742-A double-blind, randomized, placebo-controlled trial of fecal microbiota transplantation capsules (FMTC) for the treatment of diarrhea-predominant irritable bowel syndrome (IBS-D). Gastroenterology.

[ref-7] Arpaia N, Campbell C, Fan X, Dikiy S, Van Der Veeken J, deRoos P, Liu H, Cross JR, Pfeffer K, Coffer PJ, Rudensky AY (2013). Metabolites produced by commensal bacteria promote peripheral regulatory T-cell generation. Nature.

[ref-8] Artis D, Wang ML, Keilbaugh SA, He W, Brenes M, Swain GP, Knight PA, Donaldson DD, Lazar MA, Miller HRP, Schad GA, Scott P, Wu GD (2004). RELMβ/FIZZ2 is a goblet cell-specific immune-effector molecule in the gastrointestinal tract. Proceedings of the National Academy of Sciences of the United States of America.

[ref-9] Bezirtzoglou E, Tsiotsias A, Welling GW (2011). Microbiota profile in feces of breast-and formula-fed newborns by using fluorescence in situ hybridization (FISH). Anaerobe.

[ref-10] Bien J, Palagani V, Bozko P (2013). The intestinal microbiota dysbiosis and *Clostridium difficile* infection: is there a relationship with inflammatory bowel disease?. Therapeutic Advances in Gastroenterology.

[ref-11] Blaser MJ (2016). Antibiotic use and its consequences for the normal microbiome. Science.

[ref-12] Borody TJ, Campbell J (2011). Fecal microbiota transplantation: current status and future directions. Expert Review of Gastroenterology & Hepatology.

[ref-13] Borody TJ, Campbell J, Torres M, Nowak A, Leis S (2011). Reversal of idiopathic thrombocytopenic purpura [ITP] with fecal microbiota transplantation [FMT]. American Journal of Gastroenterology.

[ref-14] Borody TJ, Nowak A, Finlayson S (2012). The GI microbiome and its role in chronic fatigue syndrome: a summary of bacteriotherapy. Journal of the Australasian College of Nutritional and Environmental Medicine.

[ref-15] Borody TJ, Warren EF, Leis S, Surace R, Ashman O (2003). Treatment of ulcerative colitis using fecal bacteriotherapy. Journal of Clinical Gastroenterology.

[ref-16] Borody TJ, Warren EF, Leis SM, Surace R, Ashman O, Siarakas S (2004). Bacteriotherapy using fecal flora: toying with human motions. Journal of Clinical Gastroenterology.

[ref-17] Brandt LJ, Borody TJ, Campbell J (2011). Endoscopic fecal microbiota transplantation: first-line treatment for severe *Clostridium difficile* infection?. Journal of Clinical Gastroenterology.

[ref-18] Buffie CG, Bucci V, Stein RR, McKenney PT, Ling L, Gobourne A, No D, Liu H, Kinnebrew M, Viale A, Littmann E, Van Den Brink MRM, Jenq RR, Taur Y, Sander C, Cross JR, Toussaint NC, Xavier JB, Pamer EG (2015). Precision microbiome reconstitution restores bile acid mediated resistance to *Clostridium difficile*. Nature.

[ref-19] Buffington SA, Di Prisco GV, Auchtung TA, Ajami NJ, Petrosino JF, Costa-Mattioli M (2016). Microbial reconstitution reverses maternal diet-induced social and synaptic deficits in offspring. Cell.

[ref-20] Buie T (2015). Potential etiologic factors of microbiome disruption in autism. Clinical Therapeutics.

[ref-21] Bunyavanich S, Shen N, Grishin A, Wood R, Burks W, Dawson P, Jones SM, Leung DYM, Sampson H, Sicherer S, Clemente JC (2016). Early-life gut microbiome composition and milk allergy resolution. Journal of Allergy and Clinical Immunology.

[ref-22] Burns DA, Heap JT, Minton NP (2010). *Clostridium difficile* spore germination: an update. Research in Microbiology.

[ref-23] Cammarota G, Ianiro G, Gasbarrini A (2014). Fecal microbiota transplantation for the treatment of *Clostridium difficile* infection: a systematic review. Journal of Clinical Gastroenterology.

[ref-24] Cammarota G, Masucci L, Ianiro G, Bibbò S, Dinoi G, Costamagna G, Sanguinetti M, Gasbarrini A (2015). Randomised clinical trial: faecal microbiota transplantation by colonoscopy vs. vancomycin for the treatment of recurrent *Clostridium difficile* infection. Alimentary Pharmacology & Therapeutics.

[ref-25] Carvalho FA, Aitken JD, Vijay-Kumar M, Gewirtz AT (2012). Toll-like receptor-gut microbiota interactions: perturb at your own risk!. Annual Review of Physiology.

[ref-26] Cash HL, Whitham CV, Behrendt CL, Hooper LV (2006). Symbiotic bacteria direct expression of an intestinal bactericidal lectin. Science.

[ref-27] Chevalier C, Stojanović O, Colin DJ, Suarez-Zamorano N, Tarallo V, Veyrat-Durebex C, Rigo D, Fabbiano S, Stevanović A, Hagemann S, Montet X, Seimbille Y, Zamboni N, Hapfelmeier S, Trajkovski M (2015). Gut microbiota orchestrates energy homeostasis during cold. Cell.

[ref-28] Chong C, Bloomfield F, O'Sullivan J (2018). Factors affecting gastrointestinal microbiome development in neonates. Nutrients.

[ref-29] Chu DM, Ma J, Prince AL, Antony KM, Seferovic MD, Aagaard KM (2017). Maturation of the infant microbiome community structure and function across multiple body sites and in relation to mode of delivery. Nature Medicine.

[ref-30] Chung H, Pamp SJ, Hill JA, Surana NK, Edelman SM, Troy EB, Reading NC, Villablanca EJ, Wang S, Mora JR, Umesaki Y, Mathis D, Benoist C, Relman DA, Kasper DL (2012). Gut immune maturation depends on colonization with a host-specific microbiota. Cell.

[ref-31] Clarke G, Stilling RM, Kennedy PJ, Stanton C, Cryan JF, Dinan TG (2014). Minireview: gut microbiota: the neglected endocrine organ. Molecular Endocrinology.

[ref-32] Claud EC, Keegan KP, Brulc JM, Lu L, Bartels D, Glass E, Chang EB, Meyer F, Antonopoulos DA (2013). Bacterial community structure and functional contributions to emergence of health or necrotizing enterocolitis in preterm infants. Microbiome.

[ref-33] Cleusix V, Lacroix C, Vollenweider S, Duboux M, Le Blay G (2007). Inhibitory activity spectrum of reuterin produced by *Lactobacillus reuteri* against intestinal bacteria. BMC Microbiology.

[ref-34] Cockburn DW, Koropatkin NM (2016). Polysaccharide degradation by the intestinal microbiota and its influence on human health and disease. Journal of Molecular Biology.

[ref-35] Collado MC, Rautava S, Aakko J, Isolauri E, Salminen S (2016). Human gut colonisation may be initiated in utero by distinct microbial communities in the placenta and amniotic fluid. Scientific Reports.

[ref-36] Couturier-Maillard A, Secher T, Rehman A, Normand S, De Arcangelis A, Haesler R, Huot L, Grandjean T, Bressenot A, Delanoye-Crespin A,  Gaillot O, Schreiber S,  Lemoine Y, Ryffel B,  Hot D, Nùñez G,  Chen G,  Rosenstiel P,  Chamaillard M (2013). NOD2-mediated dysbiosis predisposes mice to transmissible colitis and colorectal cancer. Journal of Clinical Investigation.

[ref-37] Cui B, Li P, Xu L, Zhao Y, Wang H, Peng Z, Xu H, Xiang J, He Z, Zhang T, Nie Y, Wu K, Fan D, Ji G, Zhang F (2015). Step-up fecal microbiota transplantation strategy: a pilot study for steroid-dependent ulcerative colitis. Journal of Translational Medicine.

[ref-38] Cui M, Xiao H, Li Y, Zhou L, Zhao S, Luo D, Zheng Q, Dong J, Zhao Y, Zhang X, Zhang J, Lu L, Wang H, Fan S (2017a). Faecal microbiota transplantation protects against radiation-induced toxicity. EMBO Molecular Medicine.

[ref-39] Cui L, Zhao T, Hu H, Zhang W, Hua X (2017b). Association study of gut flora in coronary heart disease through high-throughput sequencing. BioMed Research International.

[ref-40] Cullen TW, Schofield WB, Barry NA, Putnam EE, Rundell EA, Trent MS, Degnan PH, Booth CJ, Yu H, Goodman AL (2015). Antimicrobial peptide resistance mediates resilience of prominent gut commensals during inflammation. Science.

[ref-41] Dahiya DK, Renuka, Puniya M, Shandilya UK, Dhewa T, Kumar N, Kumar S, Puniya AK, Shukla P (2017). Gut microbiota modulation and its relationship with obesity using prebiotic fibers and probiotics: a review. Frontiers in Microbiology.

[ref-42] Dalmasso G, Nguyen HTT, Yan Y, Laroui H, Charania MA, Ayyadurai S, Sitaraman SV, Merlin D, DeLeo FR (2011). Microbiota modulate host gene expression via microRNAs. PLOS ONE.

[ref-43] Damman C, Brittnacher M, Hayden H, Radey M, Hager K, Miller S, Zisman TL (2014). Su1403 single colonoscopically administered fecal microbiota transplant for ulcerative colitis-a pilot study to determine therapeutic benefit and graft stability. Gastroenterology.

[ref-44] David LA, Maurice CF, Carmody RN, Gootenberg DB, Button JE, Wolfe BE, Ling AV, Devlin AS, Varma Y, Fischbach MA, Biddinger SB, Dutton RJ, Turnbaugh PJ (2014). Diet rapidly and reproducibly alters the human gut microbiome. Nature.

[ref-45] Davido B, Batista R, Michelon H, Lepainteur M, Bouchand F, Lepeule R, Salomon J, Vittecoq D, Duran C, Escaut L, Sobhani I, Paul M, Lawrence C, Perronne C, Chast F, Dinh A (2017). Is faecal microbiota transplantation an option to eradicate highly drug-resistant enteric bacteria carriage?. Journal of Hospital Infection.

[ref-46] De Palma G, Lynch MDJ, Lu J, Dang VT, Deng Y, Jury J, Umeh G, Miranda PM, Pigrau Pastor M, Sidani S, Pinto-Sanchez MI, Philip V, McLean PG, Hagelsieb M-G, Surette MG, Bergonzelli GE, Verdu EF, Britz-McKibbin P, Neufeld JD, Collins SM, Bercik P (2017). Transplantation of fecal microbiota from patients with irritable bowel syndrome alters gut function and behavior in recipient mice. Science Translational Medicine.

[ref-47] De Vrese M, Marteau PR (2007). Probiotics and prebiotics: effects on diarrhea. Journal of Nutrition.

[ref-48] DeFilipp Z, Peled JU, Li S, Mahabamunuge J, Dagher Z, Slingerland AE, Del Rio C, Valles B, Kempner ME, Smith M, Brown J, Dey BR, El-Jawahri A, McAfee SL, Spitzer TR, Ballen KK, Sung AD, Dalton TE, Messina JA, Dettmer K, Liebisch G, Oefner P, Taur Y, Pamer EG, Holler E, Mansour MK, Van den Brink MRM, Hohmann E, Jenq RR, Chen Y-B (2018). Third-party fecal microbiota transplantation following allo-HCT reconstitutes microbiome diversity. Blood Advances.

[ref-49] Desai MS, Seekatz AM, Koropatkin NM, Kamada N, Hickey CA, Wolter M, Pudlo NA, Kitamoto S, Terrapon N, Muller A, Young VB, Henrissat B, Wilmes P, Stappenbeck TS, Núñez G, Martens EC (2016). A dietary fiber-deprived gut microbiota degrades the colonic mucus barrier and enhances pathogen susceptibility. Cell.

[ref-50] Dethlefsen L, Huse S, Sogin ML, Relman DA (2008). The pervasive effects of an antibiotic on the human gut microbiota, as revealed by deep 16S rRNA sequencing. PLOS Biology.

[ref-51] Dethlefsen L, Relman DA (2010). Incomplete recovery and individualized responses of the human distal gut microbiota to repeated antibiotic perturbation. Proceedings of the National Academy of Sciences of the United States of America.

[ref-52] Dicksved J, Lindberg M, Rosenquist M, Enroth H, Jansson JK, Engstrand L (2009). Molecular characterization of the stomach microbiota in patients with gastric cancer and in controls. Journal of Medical Microbiology.

[ref-53] DiGiulio DB, Romero R, Amogan HP, Kusanovic JP, Bik EM, Gotsch F, Kim CJ, Erez O, Edwin S, Relman DA, Fisk NM (2008). Microbial prevalence, diversity and abundance in amniotic fluid during preterm labor: a molecular and culture-based investigation. PLOS ONE.

[ref-54] Ding C, Fan W, Gu L, Tian H, Ge X, Gong J, Nie Y, Li N (2017). Outcomes and prognostic factors of fecal microbiota transplantation in patients with slow transit constipation: results from a prospective study with long-term follow-up. Gastroenterology Report.

[ref-55] Dinleyici EC, Eren M, Ozen M, Yargic ZA, Vandenplas Y (2012). Effectiveness and safety of *Saccharomyces boulardii* for acute infectious diarrhea. Expert Opinion on Biological Therapy.

[ref-56] Djuranovic S, Nahvi A, Green R (2012). miRNA-mediated gene silencing by translational repression followed by mRNA deadenylation and decay. Science.

[ref-57] Dominguez-Bello MG, Costello EK, Contreras M, Magris M, Hidalgo G, Fierer N, Knight R (2010). Delivery mode shapes the acquisition and structure of the initial microbiota across multiple body habitats in newborns. Proceedings of the National Academy of Sciences of the United States of America.

[ref-58] Earle KA, Billings G, Sigal M, Lichtman JS, Hansson GC, Elias JE, Amieva MR, Huang KC, Sonnenburg JL (2015). Quantitative imaging of gut microbiota spatial organization. Cell Host & Microbe.

[ref-59] Eiseman B, Silen W, Bascom GS, Kauvar AJ (1958). Fecal enema as an adjunct in the treatment of pseudomembranous enterocolitis. Surgery.

[ref-60] Everard A, Belzer C, Geurts L, Ouwerkerk JP, Druart C, Bindels LB, Guiot Y, Derrien M, Muccioli GG, Delzenne NM, De Vos WM, Cani PD (2013). Cross-talk between *Akkermansia muciniphila* and intestinal epithelium controls diet-induced obesity. Proceedings of the National Academy of Sciences of the Unites States of America.

[ref-61] Friedrich MJ (2013). Genomes of microbes inhabiting the body offer clues to human health and disease. JAMA.

[ref-62] Gao XW, Mubasher M, Fang CY, Reifer C, Miller LE (2010). Dose-response efficacy of a proprietary probiotic formula of *Lactobacillus acidophilus* CL1285 and *Lactobacillus casei* LBC80R for antibiotic-associated diarrhea and *Clostridium difficile*-associated diarrhea prophylaxis in adult patients. American Journal of Gastroenterology.

[ref-63] Ge X, Ding C, Zhao W, Xu L, Tian H, Gong J, Zhu M, Li J, Li N (2017). Antibiotics-induced depletion of mice microbiota induces changes in host serotonin biosynthesis and intestinal motility. Journal of Translational Medicine.

[ref-64] Gerassy-Vainberg S, Blatt A, Danin-Poleg Y, Gershovich K, Sabo E, Nevelsky A, Daniel S, Dahan A, Ziv O, Dheer R, Abreu MT, Koren O, Kashi Y, Chowers Y (2018). Radiation induces proinflammatory dysbiosis: transmission of inflammatory susceptibility by host cytokine induction. Gut.

[ref-65] Gibson GR, Hutkins R, Sanders ME, Prescott SL, Reimer RA, Salminen SJ, Scott K, Stanton C, Swanson KS, Cani PD, Verbeke K, Reid G (2017). Expert consensus document: The International Scientific Association for Probiotics and Prebiotics (ISAPP) consensus statement on the definition and scope of prebiotics. Nature Reviews Gastroenterology & Hepatology.

[ref-66] Gibson GR, Roberfroid MB (1995). Dietary modulation of the human colonic microbiota: introducing the concept of prebiotics. Journal of Nutrition.

[ref-67] Goldenberg RL, Hauth JC, Andrews WW (2000). Intrauterine infection and preterm delivery. New England Journal of Medicine.

[ref-68] Goodrich JK, Waters JL, Poole AC, Sutter JL, Koren O, Blekhman R, Beaumont M, Van Treuren W, Knight R, Bell JT, Spector TD, Clark AG, Ley RE (2014). Human genetics shape the gut microbiome. Cell.

[ref-69] Goyal A, Yeh A, Bush BR, Firek BA, Siebold LM, Rogers MB, Kufen AD, Morowitz MJ (2018). Safety, clinical response, and microbiome findings following fecal microbiota transplant in children with inflammatory bowel disease. Inflammatory Bowel Diseases.

[ref-70] Grehan MJ, Borody TJ, Leis SM, Campbell J, Mitchell H, Wettstein A (2010). Durable alteration of the colonic microbiota by the administration of donor fecal flora. Journal of Clinical Gastroenterology.

[ref-71] Groer MW, Luciano AA, Dishaw LJ, Ashmeade TL, Miller E, Gilbert JA (2014). Development of the preterm infant gut microbiome: a research priority. Microbiome.

[ref-72] Grönlund MM, Arvilommi H, Kero P, Lehtonen OP, Isolauri E (2000). Importance of intestinal colonisation in the maturation of humoral immunity in early infancy: a prospective follow up study of healthy infants aged 0-6 months. Archives of Disease in Childhood—Fetal and Neonatal Edition.

[ref-73] Gross L (2007). Microbes colonize a baby’s gut with distinction. PLOS Biology.

[ref-74] Guaraldi F, Salvatori G (2012). Effect of breast and formula feeding on gut microbiota shaping in newborns. Frontiers in Cellular and Infection Microbiology.

[ref-75] Halkjær SI, Christensen AH, Lo BZS, Browne PD, Günther S, Hansen LH, Petersen AM (2018). Faecal microbiota transplantation alters gut microbiota in patients with irritable bowel syndrome: results from a randomised, double-blind placebo-controlled study. Gut.

[ref-76] Hamilton MJ, Weingarden AR, Sadowsky MJ, Khoruts A (2012). Standardized frozen preparation for transplantation of fecal microbiota for recurrent *Clostridium difficile* infection. American Journal of Gastroenterology.

[ref-77] Harsch IA, Konturek PC (2019). Adhesion ileus after fecal microbiota transplantation in long-standing radiation colitis. Case Reports in Gastrointestinal Medicine.

[ref-78] He Z, Li P, Zhu J, Cui B, Xu L, Xiang J, Zhang T, Long C, Huang G, Ji G, Nie Y, Wu K, Fan D, Zhang F (2017). Multiple fresh fecal microbiota transplants induces and maintains clinical remission in Crohn’s disease complicated with inflammatory mass. Scientific Reports.

[ref-79] Hehemann J-H, Correc G, Barbeyron T, Helbert W, Czjzek M, Michel G (2010). Transfer of carbohydrate-active enzymes from marine bacteria to Japanese gut microbiota. Nature.

[ref-80] Hellemans R, Naegels S, Holvoet J (2009). Fecal transplantation for recurrent *Clostridium difficile* colitis, an underused treatment modality. Acta Gastro-Enterologica Belgica.

[ref-81] Hendijani F, Akbari V (2018). Probiotic supplementation for management of cardiovascular risk factors in adults with type II diabetes: a systematic review and meta-analysis. Clinical Nutrition.

[ref-82] Hirsch BE, Saraiya N, Poeth K, Schwartz RM, Epstein ME, Honig G (2015). Effectiveness of fecal-derived microbiota transfer using orally administered capsules for recurrent *Clostridium difficile* infection. BMC Infectious Diseases.

[ref-83] Holscher HD (2017). Dietary fiber and prebiotics and the gastrointestinal microbiota. Gut Microbes.

[ref-84] Holvoet T, Joossens M, Wang J, Boelens J, Verhasselt B, Laukens D, Van Vlierberghe H, Hindryckx P, De Vos M, De Looze D, Raes J (2017). Assessment of faecal microbial transfer in irritable bowel syndrome with severe bloating. Gut.

[ref-85] Hooper LV (2009). Do symbiotic bacteria subvert host immunity?. Nature Reviews Microbiology.

[ref-86] Hornig M (2013). The role of microbes and autoimmunity in the pathogenesis of neuropsychiatric illness. Current Opinion in Rheumatology.

[ref-87] Houghteling PD, Walker WA (2015). Why is initial bacterial colonization of the intestine important to the infant’s and child’s health?. Journal of Pediatric Gastroenterology and Nutrition.

[ref-88] Hu Z, Zhang Y, Li Z, Yu Y, Kang W, Han Y, Geng X, Ge S, Sun Y (2016). Effect of *Helicobacter pylori* infection on chronic periodontitis by the change of microecology and inflammation. Oncotarget.

[ref-89] Huebner ES, Surawicz CM (2006). Probiotics in the prevention and treatment of gastrointestinal infections. Gastroenterology Clinics of North America.

[ref-90] Ianiro G, Bibbò S, Scaldaferri F, Gasbarrini A, Cammarota G (2014). Fecal microbiota transplantation in inflammatory bowel disease: beyond the excitement. Medicine.

[ref-91] Ikram S, Hassan N, Raffat MA, Mirza S, Akram Z (2018). Systematic review and meta-analysis of double-blind, placebo-controlled, randomized clinical trials using probiotics in chronic periodontitis. Journal of Investigative and Clinical Dentistry.

[ref-92] Isaac S, Scher JU, Djukovic A, Jiménez N, Littman DR, Abramson SB, Pamer EG, Ubeda C (2016). Short-and long-term effects of oral vancomycin on the human intestinal microbiota. Journal of Antimicrobial Chemotherapy.

[ref-93] Jakobsson HE, Jernberg C, Andersson AF, Sjölund-Karlsson M, Jansson JK, Engstrand L (2010). Short-term antibiotic treatment has differing long-term impacts on the human throat and gut microbiome. PLOS ONE.

[ref-94] Jandhyala SM, Talukdar R, Subramanyam C, Vuyyuru H, Sasikala M, Reddy DN (2015). Role of the normal gut microbiota. World Journal of Gastroenterology.

[ref-95] Jernberg C, Löfmark S, Edlund C, Jansson JK (2007). Long-term ecological impacts of antibiotic administration on the human intestinal microbiota. ISME Journal.

[ref-96] Jie Z, Xia H, Zhong S-L, Feng Q, Li S, Liang S, Zhong H, Liu Z, Gao Y, Zhao H, Zhang D, Su Z, Fang Z, Lan Z, Li J, Xiao L, Li J, Li R, Li X, Li F, Ren H, Huang Y, Peng Y, Li G, Wen B, Dong B, Chen J-Y, Geng Q-S, Zhang Z-W, Yang H, Wang J, Wang J, Zhang X, Madsen L, Brix S, Ning G, Xu X, Liu X, Hou Y, Jia H, He K, Kristiansen K (2017). The gut microbiome in atherosclerotic cardiovascular disease. Nature Communications.

[ref-97] Jiménez E, Marín ML, Martín R, Odriozola JM, Olivares M, Xaus J, Fernández L, Rodríguez JM (2008). Is meconium from healthy newborns actually sterile?. Research in Microbiology.

[ref-98] Johnsen PH, Hilpüsch F, Cavanagh JP, Leikanger IS, Kolstad C, Valle PC, Goll R (2018). Faecal microbiota transplantation versus placebo for moderate-to-severe irritable bowel syndrome: a double-blind, randomised, placebo-controlled, parallel-group, single-centre trial. The Lancet Gastroenterology & Hepatology.

[ref-99] Kakihana K, Fujioka Y, Suda W, Najima Y, Kuwata G, Sasajima S, Mimura I, Morita H, Sugiyama D, Nishikawa H, Hattori M, Hino Y, Ikegawa S, Yamamoto K, Toya T, Doki N, Koizumi K, Honda K, Ohashi K (2016). Fecal microbiota transplantation for patients with steroid-resistant acute graft-versus-host disease of the gut. Blood.

[ref-100] Kalla R, Ventham NT, Kennedy NA, Quintana JF, Nimmo ER, Buck AH, Satsangi J (2014). MicroRNAs: new players in IBD. Gut.

[ref-101] Kang D-W, Adams JB, Gregory AC, Borody T, Chittick L, Fasano A, Khoruts A, Geis E, Maldonado J, McDonough-Means S, Pollard EL, Roux S, Sadowsky MJ, Lipson KS, Sullivan MB, Caporaso JG, Krajmalnik-Brown R (2017). Microbiota transfer therapy alters gut ecosystem and improves gastrointestinal and autism symptoms: an open-label study. Microbiome.

[ref-102] Karlsson F, Tremaroli V, Nielsen J, Backhed F (2013). Assessing the human gut microbiota in metabolic diseases. Diabetes.

[ref-103] Kassam Z, Hundal R, Marshall JK, Lee CH (2012). Fecal transplant via retention enema for refractory or recurrent *Clostridium difficile* infection. Archives of Internal Medicine.

[ref-104] Kassam Z, Lee CH, Yuan Y, Hunt RH (2013). Fecal microbiota transplantation for *Clostridium difficile* infection: systematic review and meta-analysis. American Journal of Gastroenterology.

[ref-105] Kelly CR, Khoruts A, Staley C, Sadowsky MJ, Abd M, Alani M, Bakow B, Curran P, McKenney J, Tisch A, Reinert SE, Machan JT, Brandt LJ (2016). Effect of fecal microbiota transplantation on recurrence in multiply recurrent *Clostridium difficile* infection: a randomized trial. Annals of Internal Medicine.

[ref-106] Kelly CP, LaMont JT (2008). *Clostridium difficile*—more difficult than ever. New England Journal of Medicine.

[ref-107] Kelly CJ, Zheng L, Campbell EL, Saeedi B, Scholz CC, Bayless AJ, Wilson KE, Glover LE, Kominsky DJ, Magnuson A, Weir TL, Ehrentraut SF, Pickel C, Kuhn KA, Lanis JM, Nguyen V, Taylor CT, Colgan SP (2015). Crosstalk between microbiota-derived short-chain fatty acids and intestinal epithelial HIF augments tissue barrier function. Cell Host & Microbe.

[ref-108] Khanna S, Pardi DS, Kelly CR, Kraft CS, Dhere T, Henn MR, Lombardo M-J, Vulic M, Ohsumi T, Winkler J, Pindar C, McGovern BH, Pomerantz RJ, Aunins JG, Cook DN, Hohmann EL (2016). A novel microbiome therapeutic increases gut microbial diversity and prevents recurrent *Clostridium difficile* infection. Journal of Infectious Diseases.

[ref-109] Khoruts A, Dicksved J, Jansson JK, Sadowsky MJ (2010). Changes in the composition of the human fecal microbiome after bacteriotherapy for recurrent *Clostridium difficile*-associated diarrhea. Journal of Clinical Gastroenterology.

[ref-110] Khoruts A, Sadowsky MJ (2016). Understanding the mechanisms of faecal microbiota transplantation. Nature Reviews Gastroenterology & Hepatology.

[ref-111] Klingensmith NJ, Coopersmith CM (2016). The gut as the motor of multiple organ dysfunction in critical illness. Critical Care Clinics.

[ref-112] Kristensen NB, Bryrup T, Allin KH, Nielsen T, Hansen TH, Pedersen O (2016). Alterations in fecal microbiota composition by probiotic supplementation in healthy adults: a systematic review of randomized controlled trials. Genome Medicine.

[ref-113] Kump P, Wurm P, Gröchenig HP, Wenzl H, Petritsch W, Halwachs B, Wagner M, Stadlbauer V, Eherer A, Hoffmann KM, Deutschmann A, Reicht G, Reiter L, Slawitsch P, Gorkiewicz G, Högenauer C (2018). The taxonomic composition of the donor intestinal microbiota is a major factor influencing the efficacy of faecal microbiota transplantation in therapy refractory ulcerative colitis. Alimentary Pharmacology & Therapeutics.

[ref-114] Kunde S, Pham A, Bonczyk S, Crumb T, Duba M, Conrad H, Cloney D, Kugathasan S (2013). Safety, tolerability, and clinical response after fecal transplantation in children and young adults with ulcerative colitis. Journal of Pediatric Gastroenterology and Nutrition.

[ref-115] Lee Y-M, Kim K-S, Jacobs DR, Lee D-H (2017). Persistent organic pollutants in adipose tissue should be considered in obesity research. Obesity Reviews.

[ref-116] Lewis BB, Buffie CG, Carter RA, Leiner I, Toussaint NC, Miller LC, Gobourne A, Ling L, Pamer EG (2015). Loss of microbiota-mediated colonization resistance to *Clostridium difficile* infection with oral vancomycin compared with metronidazole. Journal of Infectious Diseases.

[ref-117] Ley RE, Backhed F, Turnbaugh P, Lozupone CA, Knight RD, Gordon JI (2005). Obesity alters gut microbial ecology. Proceedings of the National Academy of Sciences of the United States of America.

[ref-118] Li J, Lin S, Vanhoutte PM, Woo CW, Xu A (2016a). *Akkermansia muciniphila* protects against atherosclerosis by preventing metabolic endotoxemia-induced inflammation in Apoe-/- mice. Circulation.

[ref-119] Li J, Zhao F, Wang Y, Chen J, Tao J, Tian G, Wu S, Liu W, Cui Q, Geng B, Zhang W, Weldon R, Auguste K, Yang L, Liu X, Chen L, Yang X, Zhu B, Cai J (2017). Gut microbiota dysbiosis contributes to the development of hypertension. Microbiome.

[ref-120] Li SS, Zhu A, Benes V, Costea PI, Hercog R, Hildebrand F, Huerta-Cepas J, Nieuwdorp M, Salojärvi J, Voigt AY, Zeller G, Sunagawa S, de Vos WM, Bork P (2016b). Durable coexistence of donor and recipient strains after fecal microbiota transplantation. Science.

[ref-121] Ling Z, Jin C, Xie T, Cheng Y, Li L, Wu N (2016). Alterations in the fecal microbiota of patients with HIV-1 infection: an observational study in a Chinese population. Scientific Reports.

[ref-122] Link A, Becker V, Goel A, Wex T, Malfertheiner P, Hoheisel JD (2012). Feasibility of fecal microRNAs as novel biomarkers for pancreatic cancer. PLOS ONE.

[ref-123] Liu S, Da Cunha AP, Rezende RM, Cialic R, Wei Z, Bry L, Comstock LE, Gandhi R, Weiner HL (2016). The host shapes the gut microbiota via fecal microRNA. Cell Host & Microbe.

[ref-124] Llopis M, Cassard-Doulcier AM, Boschat L, Bruneau A, Cailleux F, Rabot S, Gaudin F, Berrebi D,  Naveau S, Gérard P, Perlemuter G (2014). Intestinal dysbiosis explains inter-individual differences in susceptibility to alcoholic liver disease. Journal of Hepatology.

[ref-125] Louie TJ, Louie MR, Krulicki W, Byrne B, Ward L (2008). Home-based fecal flora infusion to arrest multiply-recurrent *Clostridium difficile* infection (CDI).

[ref-126] Luckey D, Gomez A, Murray J, White B, Taneja V (2013). Bugs & us: the role of the gut in autoimmunity. Indian Journal of Medical Research.

[ref-127] M'Rabet L, Vos AP, Boehm G̈, Garssen J (2008). Breast-feeding and its role in early development of the immune system in infants: consequences for health later in life. Journal of Nutrition.

[ref-128] Mackie RI, Sghir A, Gaskins HR (1999). Developmental microbial ecology of the neonatal gastrointestinal tract. American Journal of Clinical Nutrition.

[ref-129] Macpherson AJ, Uhr T (2004). Induction of protective IgA by intestinal dendritic cells carrying commensal bacteria. Science.

[ref-130] Makkawi S, Camara-Lemarroy C, Metz L (2018). Fecal microbiota transplantation associated with 10 years of stability in a patient with SPMS. Neurology-Neuroimmunology Neuroinflammation.

[ref-131] Massacand JC, Kaiser P, Ernst B, Tardivel A, Bürki K, Schneider P, Harris NL (2008). Intestinal bacteria condition dendritic cells to promote IgA production. PLOS ONE.

[ref-132] Mattila E, Uusitalo–Seppälä R, Wuorela M, Lehtola L, Nurmi H, Ristikankare M, Moilanen V, Salminen K, Seppälä M, Mattila PS, Anttila V–J, Arkkila P (2012). Fecal transplantation, through colonoscopy, is effective therapy for recurrent *Clostridium difficile* infection. Gastroenterology.

[ref-133] Maudet C, Mano M, Eulalio A (2014). MicroRNAs in the interaction between host and bacterial pathogens. FEBS Letters.

[ref-134] Mizuno S, Masaoka T, Naganuma M, Kishimoto T, Kitazawa M, Kurokawa S, Nakashima M, Takeshita K, Suda W, Mimura M, Hattori M, Kanai T (2017). Bifidobacterium-rich fecal donor may be a positive predictor for successful fecal microbiota transplantation in patients with irritable bowel syndrome. Digestion.

[ref-135] Moayyedi P, Surette MG, Kim PT, Libertucci J, Wolfe M, Onischi C, Armstrong D, Marshall JK, Kassam Z, Reinisch W, Lee CH (2015). Fecal microbiota transplantation induces remission in patients with active ulcerative colitis in a randomized controlled trial. Gastroenterology.

[ref-136] Mohan R, Koebnick C, Schildt J, Mueller M, Radke M, Blaut M (2008). Effects of *Bifidobacterium lactis* Bb12 supplementation on body weight, fecal pH, acetate, lactate, calprotectin, and IgA in preterm infants. Pediatric Research.

[ref-137] Mohan R, Koebnick C, Schildt J, Schmidt S, Mueller M, Possner M, Radke M, Blaut M (2006). Effects of *Bifidobacterium lactis* Bb12 supplementation on intestinal microbiota of preterm infants: a double-blind, placebo-controlled, randomized study. Journal of Clinical Microbiology.

[ref-138] Mukherjee S, Zheng H, Derebe MG, Callenberg KM, Partch CL, Rollins D, Propheter DC, Rizo J, Grabe M, Jiang Q-X, Hooper LV (2014). Antibacterial membrane attack by a pore-forming intestinal C-type lectin. Nature.

[ref-139] Nagpal R, Tsuji H, Takahashi T, Nomoto K, Kawashima K, Nagata S, Yamashiro Y (2017). Ontogenesis of the gut microbiota composition in healthy, full-term, vaginally born and breast-fed infants over the first 3 years of life: a quantitative bird’s-eye view. Frontiers in Microbiology.

[ref-140] Nishino K, Nishida A, Inoue R, Kawada Y, Ohno M, Sakai S, Inatomi O, Bamba S, Sugimoto M, Kawahara M, Naito Y, Andoh A (2018). Analysis of endoscopic brush samples identified mucosa-associated dysbiosis in inflammatory bowel disease. Journal of Gastroenterology.

[ref-141] Odamaki T, Kato K, Sugahara H, Hashikura N, Takahashi S, Xiao J-Z, Abe F, Osawa R (2016). Age-related changes in gut microbiota composition from newborn to centenarian: a cross-sectional study. BMC Microbiology.

[ref-142] Ouwehand A, Isolauri E, Salminen S (2002). The role of the intestinal microflora for the development of the immune system in early childhood. European Journal of Nutrition.

[ref-143] Paramsothy S, Kamm M, Walsh A, Van Den Bogaerde J, Douglas Samuel RL, Connor S, Ng W, Paramsothy R, Kaakoush N (2016). Multi-donor intense faecal microbiota transplantation is an effective treatment for resistant ulcerative colitis: a randomised placebo-controlled trial. Journal of Crohn's and Colitis.

[ref-144] Paterson DL, Iredell J, Whitby M (1994). Putting back the bugs: bacterial treatment relieves chronic diarrhoea. Medical Journal of Australia.

[ref-145] Pepin J, Alary M-E, Valiquette L, Raiche E, Ruel J, Fulop K, Godin D, Bourassa C (2005). Increasing risk of relapse after treatment of *Clostridium difficile* colitis in Quebec, Canada. Clinical Infectious Diseases.

[ref-146] Persky SE, Brandt LJ (2000). Treatment of recurrent *Clostridium difficile*-associated diarrhea by administration of donated stool directly through a colonoscope. American Journal of Gastroenterology.

[ref-147] Pinn DM, Aroniadis OC, Brandt LJ (2014). Is fecal microbiota transplantation the answer for irritable bowel syndrome? A single-center experience. American Journal of Gastroenterology.

[ref-148] Podolsky DK, Lynch-Devaney K, Stow JL, Oates P, Murgue B, DeBeaumont M, Sands BE, Mahida YR (1993). Identification of human intestinal trefoil factor. Goblet cell-specific expression of a peptide targeted for apical secretion. Journal of Biological Chemistry.

[ref-149] Qin J, Li R, Raes J, Arumugam M, Burgdorf KS, Manichanh C, Nielsen T, Pons N, Levenez F, Yamada T, Mende DR, Li J, Xu J, Li S, Li D, Cao J, Wang B, Liang H, Zheng H, Xie Y, Tap J, Lepage P, Bertalan M, Batto J-M, Hansen T, Le Paslier D, Linneberg A, Nielsen HB, Pelletier E, Renault P, Sicheritz-Ponten T, Turner K, Zhu H, Yu C, Li S, Jian M, Zhou Y, Li Y, Zhang X, Li S, Qin N, Yang H, Wang J, Brunak S, Doré J, Guarner F, Kristiansen K, Pedersen O, Parkhill J, Weissenbach J, Bork P, Ehrlich SD, Wang J (2010). A human gut microbial gene catalogue established by metagenomic sequencing. Nature.

[ref-150] Quraishi MN, Sergeant MJ, Kay GL, Iqbal T, Constantinidou C, Chan J, Trivedi PJ, Ferguson JW, Adams DH, Pallen MJ (2014). Probing the microbiota in PSC: the gut adherent microbiota of PSC-IBD is distinct to that of IBD and controls. Hepatology.

[ref-151] Raetz CRH, Whitfield C (2002). Lipopolysaccharide endotoxins. Annual Review of Biochemistry.

[ref-152] Ramnani P, Chitarrari R, Tuohy K, Grant J, Hotchkiss S, Philp K, Campbell R, Gill C, Rowland I (2012). In vitro fermentation and prebiotic potential of novel low molecular weight polysaccharides derived from agar and alginate seaweeds. Anaerobe.

[ref-153] Randal Bollinger R, Everett ML, Palestrant D, Love SD, Lin SS, Parker W (2003). Human secretory immunoglobulin A may contribute to biofilm formation in the gut. Immunology.

[ref-154] Rawls JF, Mahowald MA, Ley RE, Gordon JI (2006). Reciprocal gut microbiota transplants from zebrafish and mice to germ-free recipients reveal host habitat selection. Cell.

[ref-155] Ray K (2018). Gut microbiota: filling up on fibre for a healthy gut. Nature Reviews Gastroenterology & Hepatology.

[ref-156] Rees CM, Hall NJ, Fleming P, Eaton S (2017). Probiotics for the prevention of surgical necrotising enterocolitis: systematic review and meta-analysis. BMJ Paediatrics Open.

[ref-157] Ringel Y, Ringel-Kulka T (2011). The rationale and clinical effectiveness of probiotics in irritable bowel syndrome. Journal of Clinical Gastroenterology.

[ref-158] Ritchie ML, Romanuk TN (2012). A meta-analysis of probiotic efficacy for gastrointestinal diseases. PLOS ONE.

[ref-159] Rokka S, Rantamäki P (2010). Protecting probiotic bacteria by microencapsulation: challenges for industrial applications. European Food Research and Technology.

[ref-160] Rossen NG, Fuentes S, Boonstra K, D’Haens GR, Heilig HG, Zoetendal EG, De Vos WM, Ponsioen CY (2014). The mucosa-associated microbiota of PSC patients is characterized by low diversity and low abundance of uncultured Clostridiales II. Journal of Crohn's and Colitis.

[ref-161] Rossen NG, Fuentes S, Van Der Spek MJ, Tijssen JG, Hartman JHA, Duflou A, Löwenberg M, Van Den Brink GR, Mathus-Vliegen EMH, De Vos WM, Zoetendal EG, D'Haens GR, Ponsioen CY (2015). Findings from a randomized controlled trial of fecal transplantation for patients with ulcerative colitis. Gastroenterology.

[ref-162] Rothschild D, Weissbrod O, Barkan E, Kurilshikov A, Korem T, Zeevi D, Costea PI, Godneva A, Kalka IN, Bar N, Shilo S, Lador D, Vila AV, Zmora N, Pevsner-Fischer M, Israeli D, Kosower N, Malka G, Wolf BC, Avnit-Sagi T, Lotan-Pompan M, Weinberger A, Halpern Z, Carmi S, Fu J, Wijmenga C, Zhernakova A, Elinav E, Segal E (2018). Environment dominates over host genetics in shaping human gut microbiota. Nature.

[ref-163] Rubin TA, Gessert CE, Aas J, Bakken JS (2013). Fecal microbiome transplantation for recurrent *Clostridium difficile* infection: report on a case series. Anaerobe.

[ref-164] Russell SL, Finlay BB (2012). The impact of gut microbes in allergic diseases. Current Opinion in Gastroenterology.

[ref-165] Salzman NH, Underwood MA, Bevins CL (2007). Paneth cells, defensins, and the commensal microbiota: a hypothesis on intimate interplay at the intestinal mucosa. Seminars in Immunology.

[ref-166] Schroeder BO, Birchenough GMH, Ståhlman M, Arike L, Johansson MEV, Hansson GC, Bäckhed F (2018). Bifidobacteria or fiber protects against diet-induced microbiota-mediated colonic mucus deterioration. Cell Host & Microbe.

[ref-168] Schwenger EM, Tejani AM, Loewen PS (2015). Probiotics for preventing urinary tract infections in adults and children. Cochrane Database of Systematic Reviews.

[ref-169] Sekirov I, Russell SL, Antunes LCM, Finlay BB (2010). Gut microbiota in health and disease. Physiological Reviews.

[ref-170] Servin AL (2004). Antagonistic activities of lactobacilli and bifidobacteria against microbial pathogens. FEMS Microbiology Reviews.

[ref-171] Sherman MP, Zaghouani H, Niklas V (2014). Gut microbiota, the immune system, and diet influence the neonatal gut-brain axis. Pediatric Research.

[ref-172] Silverman MS, Davis I, Pillai DR (2010). Success of self-administered home fecal transplantation for chronic *Clostridium difficile* infection. Clinical Gastroenterology and Hepatology.

[ref-173] Sivan A, Corrales L, Hubert N, Williams JB, Aquino-Michaels K, Earley ZM, Benyamin FW, Man Lei Y, Jabri B, Alegre M-L, Chang EB, Gajewski TF (2015). Commensal Bifidobacterium promotes antitumor immunity and facilitates anti-PD-L1 efficacy. Science.

[ref-174] Slavin J (2013). Fiber and prebiotics: mechanisms and health benefits. Nutrients.

[ref-175] Spindelboeck W, Schulz E, Uhl B, Kashofer K, Aigelsreiter A, Zinke-Cerwenka W, Mulabecirovic A, Kump PK, Halwachs B, Gorkiewicz G, Sill H, Greinix H, Högenauer C, Neumeister P (2017). Repeated fecal microbiota transplantations attenuate diarrhea and lead to sustained changes in the fecal microbiota in acute, refractory gastrointestinal graft-versus-host-disease. Haematologica.

[ref-176] Stark PL, Lee A, Parsonage BD (1982). Colonization of the large bowel by *Clostridium difficile* in healthy infants: quantitative study. Infection and Immunity.

[ref-177] Stecher B, Hardt W-D (2008). The role of microbiota in infectious disease. Trends in Microbiology.

[ref-178] Stratiki Z, Costalos C, Sevastiadou S, Kastanidou O, Skouroliakou M, Giakoumatou A, Petrohilou V (2007). The effect of a bifidobacter supplemented bovine milk on intestinal permeability of preterm infants. Early Human Development.

[ref-179] Suskind DL, Brittnacher MJ, Wahbeh G, Shaffer ML, Hayden HS, Qin X, Singh N, Damman CJ, Hager KR, Nielson H, Miller SI (2015). Fecal microbial transplant effect on clinical outcomes and fecal microbiome in active Crohn's disease. Inflammatory Bowel Diseases.

[ref-180] Suzuki K, Meek B, Doi Y, Muramatsu M, Chiba T, Honjo T, Fagarasan S (2004). Aberrant expansion of segmented filamentous bacteria in IgA-deficient gut. Proceedings of the National Academy of Sciences of the United States of America.

[ref-181] Tabibian JH, Talwalkar JA, Lindor KD (2013). Role of the microbiota and antibiotics in primary sclerosing cholangitis. BioMed Research International.

[ref-182] Tailford LE, Crost EH, Kavanaugh D, Juge N (2015). Mucin glycan foraging in the human gut microbiome. Frontiers in Genetics.

[ref-183] Takahashi K, Nishida A, Fujimoto T, Fujii M, Shioya M, Imaeda H, Inatomi O, Bamba S, Andoh A, Sugimoto M (2016). Reduced abundance of butyrate-producing bacteria species in the fecal microbial community in Crohn's disease. Digestion.

[ref-184] Takeuchi O, Akira S (2010). Pattern recognition receptors and inflammation. Cell.

[ref-185] Tang WHW, Wang Z, Levison BS, Koeth RA, Britt EB, Fu X, Wu Y, Hazen SL (2013). Intestinal microbial metabolism of phosphatidylcholine and cardiovascular risk. New England Journal of Medicine.

[ref-186] Tannock GW, Munro K, Harmsen HJM, Welling GW, Smart J, Gopal PK (2000). Analysis of the fecal microflora of human subjects consuming a probiotic product containing *Lactobacillus rhamnosus* DR20. Applied and Environmental Microbiology.

[ref-187] Theriot CM, Koenigsknecht MJ, Carlson PE, Hatton GE, Nelson AM, Li B, Huffnagle GB, Li JZ, Young VB (2014). Antibiotic-induced shifts in the mouse gut microbiome and metabolome increase susceptibility to *Clostridium difficile* infection. Nature Communications.

[ref-188] Thursby E, Juge N (2017). Introduction to the human gut microbiota. Biochemical Journal.

[ref-189] Tian H, Ding C, Gong J, Wei Y, McFarland LV, Li N (2015). Freeze-dried, capsulized fecal microbiota transplantation for relapsing *Clostridium difficile* infection. Journal of Clinical Gastroenterology.

[ref-190] Tian H, Ge X, Nie Y, Yang L, Ding C, McFarland LV, Zhang X, Chen Q, Gong J, Li N, Green J (2017). Fecal microbiota transplantation in patients with slow-transit constipation: a randomized, clinical trial. PLOS ONE.

[ref-191] Tilg H, Kaser A (2011). Gut microbiome, obesity, and metabolic dysfunction. Journal of Clinical Investigation.

[ref-192] Tremaroli V, Bäckhed F (2012). Functional interactions between the gut microbiota and host metabolism. Nature.

[ref-193] Trøseid M (2017). Gut microbiota and acute coronary syndromes: ready for use in the emergency room?. European Heart Journal.

[ref-194] Turnbaugh PJ, Hamady M, Yatsunenko T, Cantarel BL, Duncan A, Ley RE, Sogin ML, Jones WJ, Roe BA, Affourtit JP, Egholm M, Henrissat B, Heath AC, Knight R, Gordon JI (2009). A core gut microbiome in obese and lean twins. Nature.

[ref-195] Udayappan SD, Hartstra AV, Dallinga-Thie GM, Nieuwdorp M (2014). Intestinal microbiota and faecal transplantation as treatment modality for insulin resistance and type 2 diabetes mellitus. Clinical & Experimental Immunology.

[ref-196] Umu ÖCO, Rudi K, Diep DB (2017). Modulation of the gut microbiota by prebiotic fibres and bacteriocins'. Microbial Ecology in Health and Disease.

[ref-197] Uygun A, Ozturk K, Demirci H, Oger C, Avci IY, Turker T, Gulsen M (2017). Fecal microbiota transplantation is a rescue treatment modality for refractory ulcerative colitis. Medicine.

[ref-198] Van Den Abbeele P, Taminiau B, Pinheiro I, Duysburgh C, Jacobs H, Pijls L, Marzorati M (2018). Arabinoxylo-oligosaccharides and inulin impact inter-individual variation on microbial metabolism and composition, which immunomodulates human cells. Journal of Agricultural and Food Chemistry.

[ref-199] Van Nood E, Dijkgraaf MG, Nieuwdorp M, Fuentes S, Zoetendal EG, De Vos WM,  Visser CE, Kuijper EJ, Bartelsman JFWM, Tijssen JGP, Speelman P, Dijkgraaf MGW, Keller JJ (2013). Duodenal infusion of feces for recurrent *Clostridium difficile*. New England Journal of Medicine.

[ref-200] Vandenplas Y, Zakharova I, Dmitrieva Y (2015). Oligosaccharides in infant formula: more evidence to validate the role of prebiotics. British Journal of Nutrition.

[ref-201] Vermeire S, Joossens M, Verbeke K, Wang J, Machiels K, Sabino J, Ferrante M, Van Assche G, Rutgeerts P, Raes J (2015). Donor species richness determines faecal microbiota transplantation success in inflammatory bowel disease. Journal of Crohn's and Colitis.

[ref-202] Vernocchi P, Del Chierico F, Putignani L (2016). Gut microbiota profiling: metabolomics based approach to unravel compounds affecting human health. Frontiers in Microbiology.

[ref-203] Vrieze A, Out C, Fuentes S, Jonker L, Reuling I, Kootte RS, Van Nood E, Holleman F, Knaapen M, Romijn JA, Soeters MR, Blaak EE, Dallinga-Thie GM, Reijnders D, Ackermans MT, Serlie MJ, Knop FK, Holst JJ, Van der Ley C, Kema IP, Zoetendal EG, De Vos WM, Hoekstra JBL, Stroes ES, Groen AK, Nieuwdorp M (2014). Impact of oral vancomycin on gut microbiota, bile acid metabolism, and insulin sensitivity. Journal of Hepatology.

[ref-204] Vrieze A, Van Nood E, Holleman F, Salojärvi J, Kootte RS, Bartelsman JFWM, Dallinga–Thie GM, Ackermans MT, Serlie MJ, Oozeer R, Derrien M, Druesne A, Van Hylckama Vlieg JET, Bloks VW, Groen AK, Heilig HGHJ, Zoetendal EG, Stroes ES, De Vos WM, Hoekstra JBL, Nieuwdorp M (2012). Transfer of intestinal microbiota from lean donors increases insulin sensitivity in individuals with metabolic syndrome. Gastroenterology.

[ref-205] Wahlström A, Sayin SI, Marschall H-U, Bäckhed F (2016). Intestinal crosstalk between bile acids and microbiota and its impact on host metabolism. Cell Metabolism.

[ref-206] Walker AW, Ince J, Duncan SH, Webster LM, Holtrop G, Ze X, Brown D, Stares MD, Scott P, Bergerat A, Louis P, McIntosh F, Johnstone AM, Lobley GE, Parkhill J, Flint HJ (2011). Dominant and diet-responsive groups of bacteria within the human colonic microbiota. ISME Journal.

[ref-207] Walker WA, Iyengar RS (2014). Breast milk, microbiota, and intestinal immune homeostasis. Pediatric Research.

[ref-208] Wampach L, Heintz-Buschart A, Hogan A, Muller EEL, Narayanasamy S, Laczny CC, Hugerth LW, Bindl L, Bottu J, Andersson AF, De Beaufort C, Wilmes P (2017). Colonization and succession within the human gut microbiome by archaea, bacteria, and microeukaryotes during the first year of life. Frontiers in Microbiology.

[ref-209] Watson AJM, Hall LJ (2013). Regulation of host gene expression by gut microbiota. Gastroenterology.

[ref-210] Weber JA, Baxter DH, Zhang S, Huang DY, How Huang K, Jen Lee M, Galas DJ, Wang K (2010). The microRNA spectrum in 12 body fluids. Clinical Chemistry.

[ref-211] Wen L, Duffy A (2017). Factors influencing the gut microbiota, inflammation, and type 2 diabetes. Journal of Nutrition.

[ref-212] Wettstein A, Borody TJ, Leis S, Chongnan J, Torres M, Hills LA (2007). Fecal bacteriotherapy-an effective treatment for relapsing symptomatic Clostridium difficile infection. Gut.

[ref-213] Wilcox GM (2011). Early experience with a fecal bacteriotherapy (FB) program for recurrent and C-difficile infection (CDI). Gastroenterology.

[ref-214] Wiley NC, Dinan TG, Ross RP, Stanton C, Clarke G, Cryan JF (2017). The microbiota-gut-brain axis as a key regulator of neural function and the stress response: implications for human and animal health. Journal of Animal Science.

[ref-215] Windey K, De Preter V, Verbeke K (2012). Relevance of protein fermentation to gut health. Molecular Nutrition & Food Research.

[ref-216] Wong SH, Zhao L, Zhang X, Nakatsu G, Han J, Xu W, Xiao X, Kwong TNY, Tsoi H, Wu WKK, Zeng B, Chan FKL, Sung JJY, Wei H, Yu J (2017). Gavage of fecal samples from patients with colorectal cancer promotes intestinal carcinogenesis in germ-free and conventional mice. Gastroenterology.

[ref-217] Wu Y, Zhang Q, Ren Y, Ruan Z, Norata GD (2017). Effect of probiotic Lactobacillus on lipid profile: a systematic review and meta-analysis of randomized, controlled trials. PLOS ONE.

[ref-218] Xie HY, Feng D, Wei DM, Mei L, Chen H, Wang X, Fang F (2017). Probiotics for vulvovaginal candidiasis in non-pregnant women. Cochrane Database of Systematic Reviews.

[ref-219] Xue X, Feng T, Yao S, Wolf KJ, Liu C-G, Liu X, Elson CO, Cong Y (2011). Microbiota downregulates dendritic cell expression of miR-10a, which targets IL-12/IL-23p40. Journal of Immunology.

[ref-220] Yang L, Lu X, Nossa CW, Francois F, Peek RM, Pei Z (2009). Inflammation and intestinal metaplasia of the distal esophagus are associated with alterations in the microbiome. Gastroenterology.

[ref-221] Yatsunenko T, Rey FE, Manary MJ, Trehan I, Dominguez-Bello MG, Contreras M, Magris M, Hidalgo G, Baldassano RN, Anokhin AP, Heath AC, Warner B, Reeder J, Kuczynski J, Caporaso JG, Lozupone CA, Lauber C, Clemente JC, Knights D, Knight R, Gordon JI (2012). Human gut microbiome viewed across age and geography. Nature.

[ref-222] Yoshioka H, Iseki K-I, Fujita K (1983). Development and differences of intestinal flora in the neonatal period in breast-fed and bottle-fed infants. Pediatrics.

[ref-223] Youngster I, Russell GH, Pindar C, Ziv-Baran T, Sauk J, Hohmann EL (2014a). Oral, capsulized, frozen fecal microbiota transplantation for relapsing *Clostridium difficile* infection. JAMA.

[ref-224] Youngster I, Sauk J, Pindar C, Wilson RG, Kaplan JL, Smith MB, Alm EJ, Gevers D, Russell GH, Hohmann EL (2014b). Fecal microbiota transplant for relapsing *Clostridium difficile* infection using a frozen inoculum from unrelated donors: a randomized, open-label, controlled pilot study. Clinical Infectious Diseases.

[ref-225] Zar FA, Bakkanagari SR, Moorthi KMLST, Davis MB (2007). A comparison of vancomycin and metronidazole for the treatment of *Clostridium difficile*-associated diarrhea, stratified by disease severity. Clinical Infectious Diseases.

[ref-226] Zarepour M, Bhullar K, Montero M, Ma C, Huang T, Velcich A, Xia L, Vallance BA, Bäumler AJ (2013). The mucin Muc2 limits pathogen burdens and epithelial barrier dysfunction during Salmonella enterica serovar *Typhimurium colitis*. Infection and Immunity.

[ref-227] Zhang F, Luo W, Shi Y, Fan Z, Ji G (2012). Should we standardize the 1,700-year-old fecal microbiota transplantation?. American Journal of Gastroenterology.

[ref-231] Zhang Q, Wu Y, Fei X (2016). Effect of probiotics on glucose metabolism in patients with type 2 diabetes mellitus: a meta-analysis of randomized controlled trials. Medicina.

[ref-228] Zhao S, Liu W, Wang J, Shi J, Sun Y, Wang W, Ning G, Liu R, Hong J (2017). *Akkermansia muciniphila* improves metabolic profiles by reducing inflammation in chow diet-fed mice. Journal of Molecular Endocrinology.

[ref-229] Zheng P, Zeng B, Liu M, Chen J, Pan J, Han Y, Liu Y, Cheng K, Zhou C, Wang H, Zhou X, Gui S, Perry SW, Wong M-L, Licinio J, Wei H, Xie P (2019). The gut microbiome from patients with schizophrenia modulates the glutamate-glutamine-GABA cycle and schizophrenia-relevant behaviors in mice. Science Advances.

[ref-230] Zhou K (2017). Strategies to promote abundance of *Akkermansia muciniphila*, an emerging probiotics in the gut, evidence from dietary intervention studies. Journal of Functional Foods.

